# *RcMYB8* enhances salt and drought tolerance in rose (*Rosa chinensis*) by modulating *RcPR5/1* and *RcP5CS1*

**DOI:** 10.1186/s43897-024-00080-9

**Published:** 2024-01-29

**Authors:** Yichang Zhang, Shuang Yu, Pengfei Niu, Lin Su, Xuecheng Jiao, Xiuyu Sui, Yaru Shi, Boda Liu, Wanpei Lu, Hong Zhu, Xinqiang Jiang

**Affiliations:** 1 College of Landscape Architecture and Forestry, Qingdao Agricultural University, Qingdao, 266109 Shandong China; 2 College of Agronomy, Qingdao Agricultural University, Qingdao, 266109 Shandong China

**Keywords:** Rose, Drought tolerance, Salinity tolerance, *RcMYB8*, *RcPR5/1*, *RcP5CS1*

## Abstract

Plant Myeloblastosis (MYB) proteins function crucially roles upon variegated abiotic stresses. Nonetheless, their effects and mechanisms in rose (*Rosa chinensis*) are not fully clarified. In this study, we characterized the effects of rose *RcMYB8* under salt and drought tolerances. For induction of the *RcMYB8* expression, NaCl and drought stress treatment were adopted. Rose plants overexpressing *RcMYB8* displayed enhanced tolerance to salinity and drought stress, while silencing *RcMYB8* resulted in decreased tolerance, as evidenced by lowered intra-leaf electrolyte leakage and callose deposition, as well as photosynthetic sustainment under stressed conditions. Here, we further show that RcMYB8 binds similarly to the promoters of *RcPR5/1* and *RcP5C51* in vivo and in vitro. Inhibiting *RcP5CS1* by virus-induced gene silencing led to decreased drought tolerance through the reactive oxygen species (ROS) homeostatic regulation. *RcP5CS1-*silenced plants showed an increase in ion leakage and reduce of proline content, together with the content of malondialdehyde (MDA) increased, lowered activities of Catalase (CAT), peroxidase (POD) and superoxide dismutase (SOD). Our study highlights the transcriptional modulator role of *RcMYB8* in drought and salinity tolerances, which bridges *RcPR5/1* and *RcP5CS1* by promoting ROS scavenging. Besides, it is probably applicable to the rose plant engineering for enhancing their abiotic stress tolerances.

**Graphical Abstract:**

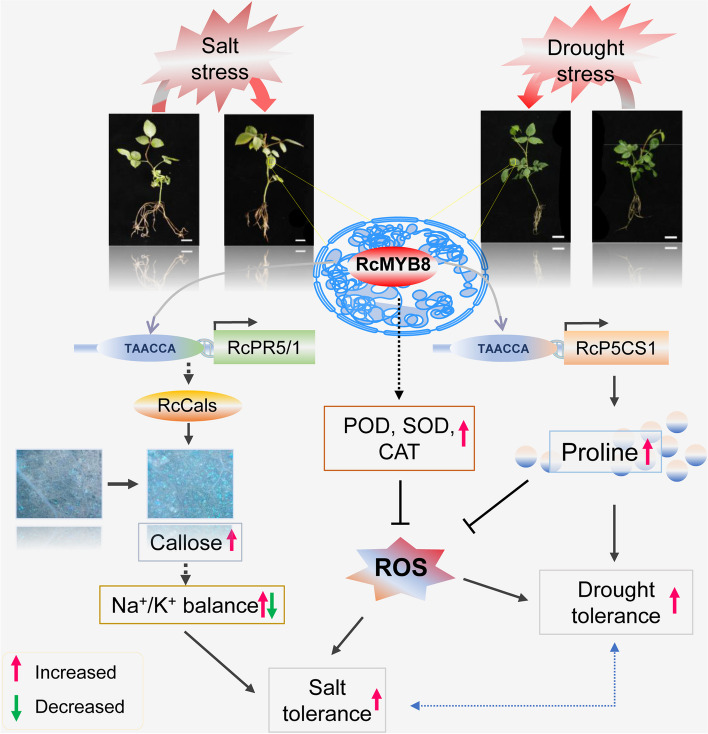

**Supplementary Information:**

The online version contains supplementary material available at 10.1186/s43897-024-00080-9.

## Core

The expression of transcription factors *RcMYB8* increased under salt and drought stress, regulating the expression of *RcPR5/1* and *RcP5CS1*. It not only influences the Na^+^/K^+^ balance through callose accumulation to enhance resistance to salt stress but also affects proline accumulation, thereby improving drought tolerance.

## Gene and accession numbers

Gene sequence information from this article can be found in the database of the National Center for Biotechnology Information (NCBI) under the accession numbers: *RcMYB8*: PRQ28032.1, *RcPR5/1*: PRQ27222.1, *RcP5CS1*: PRQ43126.1, *RcPR4/1*: PRQ41818.1, *AtPAP1*: AAG42001, *AtPAP2*: AAG42002, *VvMYBA1*: BAD18977.1, *SbMYB8*: KF008657.1, *LhMYB6*: BAJ05399, *OsMYB4*: D88620.1, *IbMYB308*: CAA2964916.1, *VvMYBPA2*: ACK56131, *VvMYBPA1*: CAJ90831.1, *PtrMYB134*: ACR83705.1, *AtMYB96*: *AJ011669.1, GhMYB36*: ASH96785.1, *AtMYB2*: AEC10812.1, *ZmMYB48*: XM_008666380.2, *AtMYB44*: Q9FDW1.1, *OsMYB3R-2*: NP_001393248.1, *TaMYB3R-1*: HQ236494.1, *AtMYB3R-1*: AF151646, *AtMYB3R-4*: Q94FL9.1, *OsMYB3R-1*: AJ430051.1, *AtMYB4R*: AY033827, *PbMYB4R*: KX272615.1, *AtLHY*: Q6R0H1.2, *AtCCA1*: P92973.1, *GmMYB118*: NP_001235909.2, *AtMYBD*: AAP21221.1, *GhMYB176*: NP_001236048.2, *HvMCB1*: CAC24844.1, *HvMCB2*: CAC24845.1, *RcP5CS2*: PRQ43126.1, *RcUBI2*: PRQ58461.1.

## Introduction

Plants often face various adverse environmental conditions during their lifestyle, which limit their growth and development. Drought and salinity constitute two chief abiotic stresses with a serious influence over plant growth and agricultural production (Zhang et al. 2022). Increased soil salt content causes ionic toxicity and oxidative stress in plants (Yang and Guo 2018). It also disrupts the balance of Na^+^/K^+^ in cells, leading to the disorder of plant cell metabolism (Munns and Tester 2008). When plants are under drought stress, the cells and tissues shrink, collapse, and grow slowly due to the lack of water, affecting their normal growth and development and further leading to loss of biomass and productivity or even death when stressed by persistent serious drought (Eziz et al. 2017).

Upon subjection to salinity and drought stress, a range of plant genes respond quickly, and their products are responsible for the expression regulation of downstream genes to exert a plant protective role by preventing or reducing stress damage (Van Zelm et al. 2020). One set of response genes consists of functional genes that directly enhance resistance through the synthesis of osmoregulatory substances, soluble sugar, proline, etc. These genes include pyrroline-5-carboxylate synthase (P5CS) (Yoshiba et al. 1995; Du et al. 2023), late embryogenesis abundant (LEA) (Xiao et al. 2007; Yu et al. 2016), and pathogenesis related (PR) (Srivastava et al. 2004; Hashimoto et al. 2004 ). The rest are transcription factor (TF), which can regulate gene expression and signal transduction under salinity or drought stress, such as NAM, ATAF1,2, CUC2 (NAC) (Shah et al. 2013; Mao et al. 2022), APETALA2/Ethylene-responsive factor (AP2/ERF) (Tang et al. 2017; Lv et al. 2020), basic/helix-loop-helix (bHLH) (Mao et al. 2017; Jiang et al. 2019), basic leucine zipper (bZIP) (Zhu et al. 2018; Bi et al. 2021), and myeloblastosis (MYB) (Wang et al. 2014 ; Du et al. 2018).

As one prevailing TF class of plants, MYB contains a DNA-binding domain in its N-terminus that is highly conserved, which encodes proteins consisting of 51–53 amino acids (aa). According to the unit repetition quantity within the MYB domain, it can be categorized under 4 major groups, namely 1R-MYB/MYB-associated, 2R-MYB (R2R3-MYB), 3R-MYB (R1R2R3-MYB), as well as 4R-MYB (Dubos et al. 2010). As demonstrated by growing evidence, MYBs are implicated in both plant development and responses to abiotic stresses. For example, *Arabidopsis AtMYB96* improves drought resistance through the ABA-dependent activation of biosynthesis of keratin wax (Seo et al. 2011). Overexpression of apple *MdSIMYB* leads to the development of stronger roots and improves the drought, cold and salinity tolerances (Wang et al. 2014). Overexpression of *OsMYB6* increases drought and salinity stress in transgenic rice (Tang et al. 2019). In addition, different MYB transcriptional regulation modules have been revealed in many plants. For example, in *Arabidopsis*, the histone-binding proteins ENAP1 and ENAP2 can interact with MYB44 to form a complex to improve tolerance to drought (Zhao et al. 2022). In *Betula platyphylla*, *BpMYB123* regulates the plentiful protein BpLEA14, consequently improving tolerance to drought (Lv et al. 2021). *GhMYB36* in cotton (*Gossypium hirsutum*) endows cotton with stronger drought resistance by enhancing the expression of the *PR1* gene (Liu et al. 2022a). As suggested by the foregoing studies, MYB has potential as a vital target for helping plants better tolerate abiotic stress.

Rose (*Rosa* spp.) is a woody plant that is widely used as indoor and outdoor landscape plants all over the world and has high economic value in the flower industry. However, in cultivation and outdoor application, roses easily suffer from drought and salinity stress, which restrict their growth and development, resulting in lower productivity, especially in saline or semiarid areas. Although MYB TFs have been studied in most model plant species, there are few studies in rose (Han et al. 2019), and most studies on MYBs in rose have focused on disease resistance (Ren et al. 2020), proanthocyanidin synthesis and anthocyanin synthesis (Li et al. 2019). The function of rose MYB genes challenged by abiotic stresses has rarely been understood.

In the present work, a novel R2R3-type MYB TF, *RcMYB8*, which was responsive to salinity and drought stress, was characterized. For the induction of *RcMYB8*, drought and salt stresses were adopted. Downregulation of *RcMYB8* using virus-induced gene silencing (VIGS) led to compromised salt and drought tolerance in *RcMYB8*-silenced plants. *RcMYB8* overexpression improved salinity and drought tolerance. We used yeast one-hybrid (Y1H), firefly luciferase/renilla luciferase (LUC/REN) and electrophoretic mobility shift assay (EMSA) analyses to show direct binding of RcMYB8 to *RcPR5/1* and *RcP5CS1* promoters’ MYB *cis*-element, thus improving salt and drought tolerance in pSuper:*RcMYB8* plants. It was also found that *RcP5CS1*-silenced plants mediated drought tolerance by influencing ROS scavenging enzyme activities. Overall, this study highlights the role of *RcMYB8* as a drought and salinity tolerance modulator in rose, provides new insights into the mechanism by coordinately regulating *RcPR5/1* and *RcP5CS1*, and has broad importance for engineering roses with enhanced resistance against abiotic stresses.

## Results

### Identification of *RcMYB8*

**Fig. 1:**
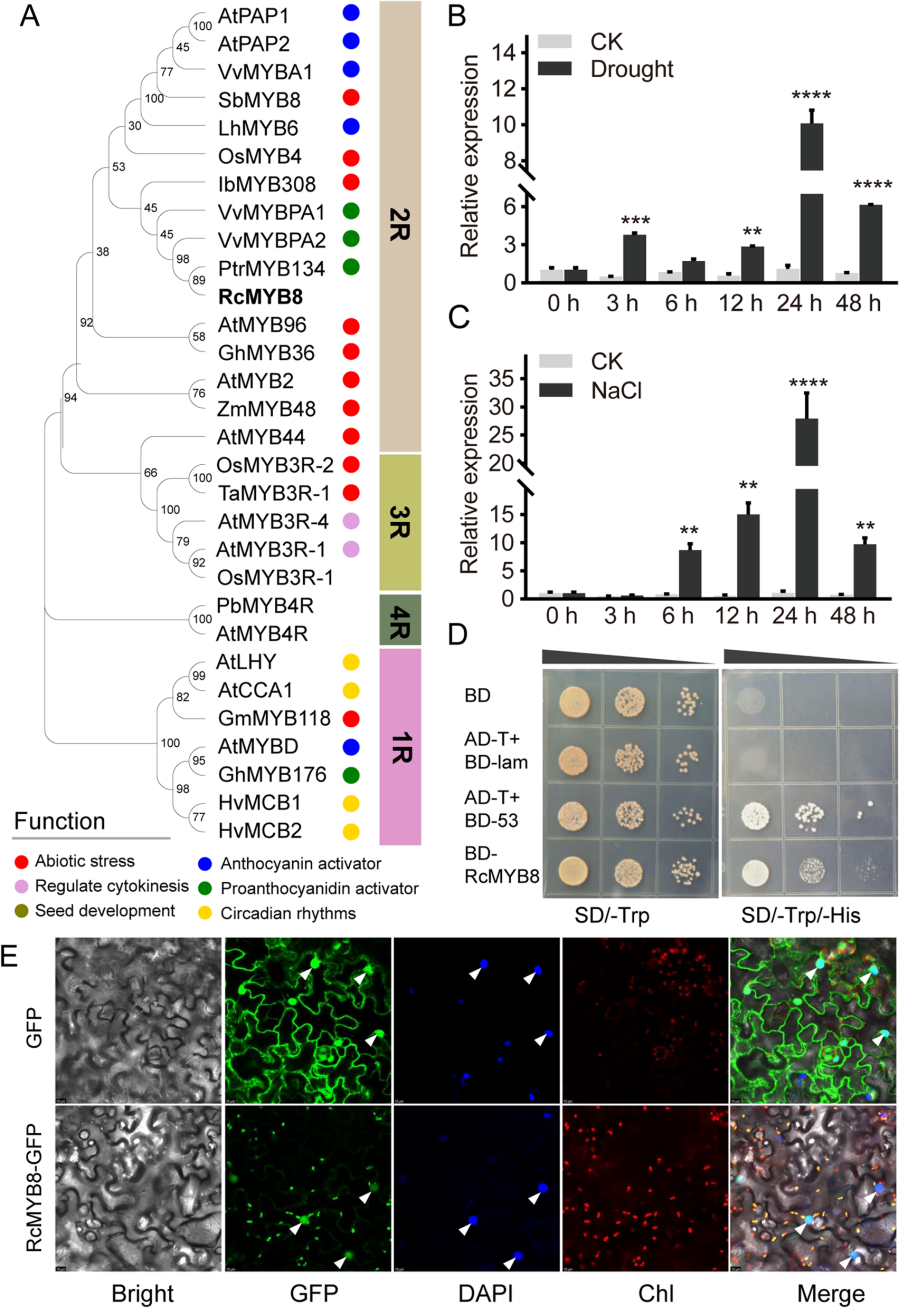
Analysis of the phytogenic, expression pattern and transactivation of *RcMYB8.* **A** Phylogenic analysis of RcMYB8 and other MYB proteins. The phylogenetic tree was constructed using the neighbor-joining method using MEGA X, with 1000 bootstrap analysis. Accession numbers for these proteins are listed in Table S2. The circle with different colors on the right illustrates the reported biological function of MYBs. **B** Expression profiles of *RcMYB8* under drought stress conditions at the indicated time points. The data originated from the three biological replicates of each treatment. **C** Expression profiles of *RcMYB8* under salt stress conditions at the indicated time points. The data originated from the three biological replicates of each treatment. Asterisks indicate statistically significant differences, as determined by Student’s *t* test (*P < 0.05; **P < 0.01; ***P < 0.001; ****P < 0.0001). **D** Transcriptional activation of *RcMYB8* in a yeast assay. Transformants harboring pGBKT7 (BD), negative control (BD + pGADT7(AD)), positive control (AD-T + BD-53), and BD-RcMYB8 were spared onto SD medium lacing Trp (SD/−Trp) or lacing Trp/His (SD/−Trp/−His) and cultured for 3 days. **E** Subcellular localization of RcMYB8. GFP and RcMYB8-GFP signals expressed in tobacco and imaged by laser confocal microscopy. The white triangle indicates the nuclear localization. Scale bar: 10 μm. GFP, Green fluorescent protein; DAPI, 4′,6-diamidino-2-phenylindole

Previous studies showed that *RcMYB8* was significantly induced in both roots and leaves of rose when challenged by drought (Li et al. 2021). We firstly isolated *RcMYB8* using specific primers (Supplemental Table. S1). The full length of *RcMYB8* is 837 bp, which encodes 278 amino acids (aa). Phylogenetic analysis revealed that RcMYB8 has high homology with *Arabidopsis* AtMYB96 (Seo et al. 2011), *Gossypium hirsutum* GhMYB36 (Liu et al. 2022a), and *Zea mays* ZmMYB48 (Wang et al. 2017), which all have similar roles in the response to abiotic stresses (Fig. 1A). Sequence alignment with RcMYB8 and seven other MYB proteins (LhMYB6, OsMYB4, SbMYB8, AtPAP1, AtPAP2, PtrMYB134, and VvMYBPA2) revealed that these proteins belong to R2R3-MYBs, which have conserved MYB domains in N-terminus and nonconserved variable domains in C-terminus. A conserved [D/E]LX2[R/K]X3LX6LX3R motif was encompassed in the sequence’s N-terminal zone, which acted as the site for bHLH protein interplays (Fig. S1).

As a next step, the expression patterns of *RcMYB8* upon drought and salinity challenges were assessed at various points of time (0, 3, 6, 12, 24, and 48 h). Compared with the expression in the control, *RcMYB8* was significantly induced to different degrees under drought conditions for 3 h (3.66-fold), 6 h (1.66-fold), 12 h (2.78-fold), 24 h (9.76-fold), and 48 h (5.94-fold) (Fig. 1B). Under salinity stress, *RcMYB8* did not increase intensively from 0 h to 3 h, while higher expressions were noted at 6 h (8.4-fold) and 12 h (14.53-fold) and the highest level was reached at 24 h (27-fold) (Fig. 1C). In addition, we measured the transactivation of RcMYB8. As is clear from Fig. 1D, the entire transformants exhibited preferable growth on tryptophan (Trp)-free synthetic dropout (SD) medium. Moreover, yeast colonies of the positive control and BD-RcMYB8 grew normally under SD conditions lacking histidine (His) and Trp (SD/−Trp/−His), implying that RcMYB8 is a transactivator. We next conducted the subcellular localization analysis RcMYB8 (Fig. 1E). The RcMYB8-green fluorescent protein (GFP) signal distribution was noted in tobacco leave nuclear cells, agreeing with its role as a TF. As suggested by the foregoing findings, RcMYB8 is a nucleus localized transcriptional activator.

### *RcMYB8* silencing decreased salt tolerance in rose

**Fig. 2:**
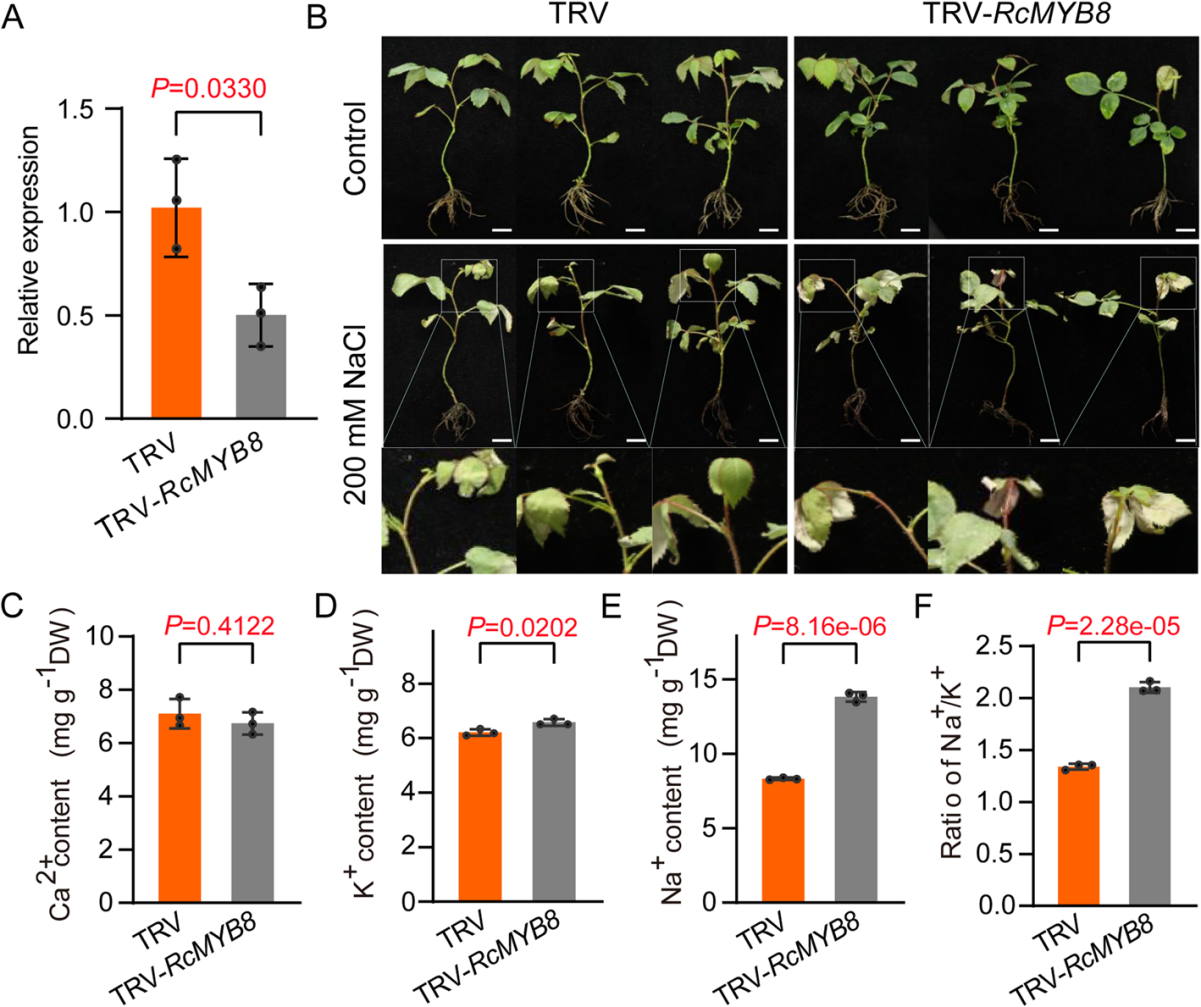
Decreased salinity tolerance of *RcMYB8*-silenced rose plants. **A** Relative expression of *RcMYB8* in TRV and TRV-*RcMYB8*. *RcUBI2* was used as an internal control of three biological replicates. **B** Performance of TRV and TRV-*RcMYB8* in rose seedlings under normal and salinity stress conditions. Nine-week-old rose plants were subjected to 0 and 200 mM NaCl for 3 days. Scale bar: 1 cm. **C** to **F** Ca^2+^ content (**C**), K^+^ content (**D**), Na^+^ content (**E**), and Na^+^/K^+^ ratio (**F**) in TRV and TRV-*RcMYB8* plants after 200 mM NaCl treatment. Data are the mean ± SD (*n* = 3)

To assess *RcMYB8* biofunction, the VIGS approach was adopted to silence it in rose plants. We firstly silenced *RcMYB8* in rose leaves that were treated normally and with NaCl (200 mM) (Fig. S2A). Compared to the tobacco rattle virus (TRV) control, TRV-*RcMYB8* exhibited 54% lower expression level of *RcMYB8* (Fig. S2B), as well as prominently higher electrolyte leakage (Fig. S2C), implying that cell membrane leakage occurred quickly when *RcMYB8* was silenced. Moreover, the chlorophyll (Chl) content in TRV-*RcMYB8* was significantly lower (1073 μg·g^−1^) than that in the TRV control (1593 μg·g^−1^) (Fig. S2D). The results of NBT and DAB staining showed that TRV-*RcMYB8* accumulated more brown and blue colors than TRV (Fig. S2E), suggesting that *RcMYB8* influenced the ROS levels when treated with salinity stress. We further silenced *RcMYB8* in rose seedlings treated for 3 d normally and with NaCl (200 mM). The *RcMYB8* level in TRV-*RcMYB8* decreased to 50% of the control group (Fig. 2A). Compared to TRV, the leaves of TRV-*RcMYB8* showed more curled and shrinkage, implying TRV-*RcMYB8* suffered more damage than TRV (Fig. 2B).

Next, the Na^+^, K^+^, and Ca^2+^ contents in TRV-*RcMYB8* and TRV were assessed as well. The inter-group difference in Ca^2+^ content was found to be insignificant (Fig. 2C)*.* The K^+^ content was slightly higher in TRV-*RcMYB8* than in TRV. With a value of 8.31 mg·g^−1^, TRV contained pronouncedly lower Na^+^ compared to the TRV-*RcMYB8* (13.83 mg·g^−1^) (Fig. 2D, E). The balance of Na^+^/K^+^ was identified as the pivotal factor upon salinity challenge (Li et al. 2023). When stressed by salinity, the Na^+^/K^+^ ratio in TRV was 1.34, showing a markedly lower value in comparison to TRV-*RcMYB8* (2.1) (Fig. 2F). Overall, silencing *RcMYB8* in rose causes broader peroxidation of membrane lipids and weakened stability of cellular membranes upon salinity challenge, and disrupts the Na^+^/K^+^ balance by increasing the Na^+^ content, resulting in decreased salinity tolerance.

### Overexpressing *RcMYB8* improved salinity tolerance

**Fig. 3:**
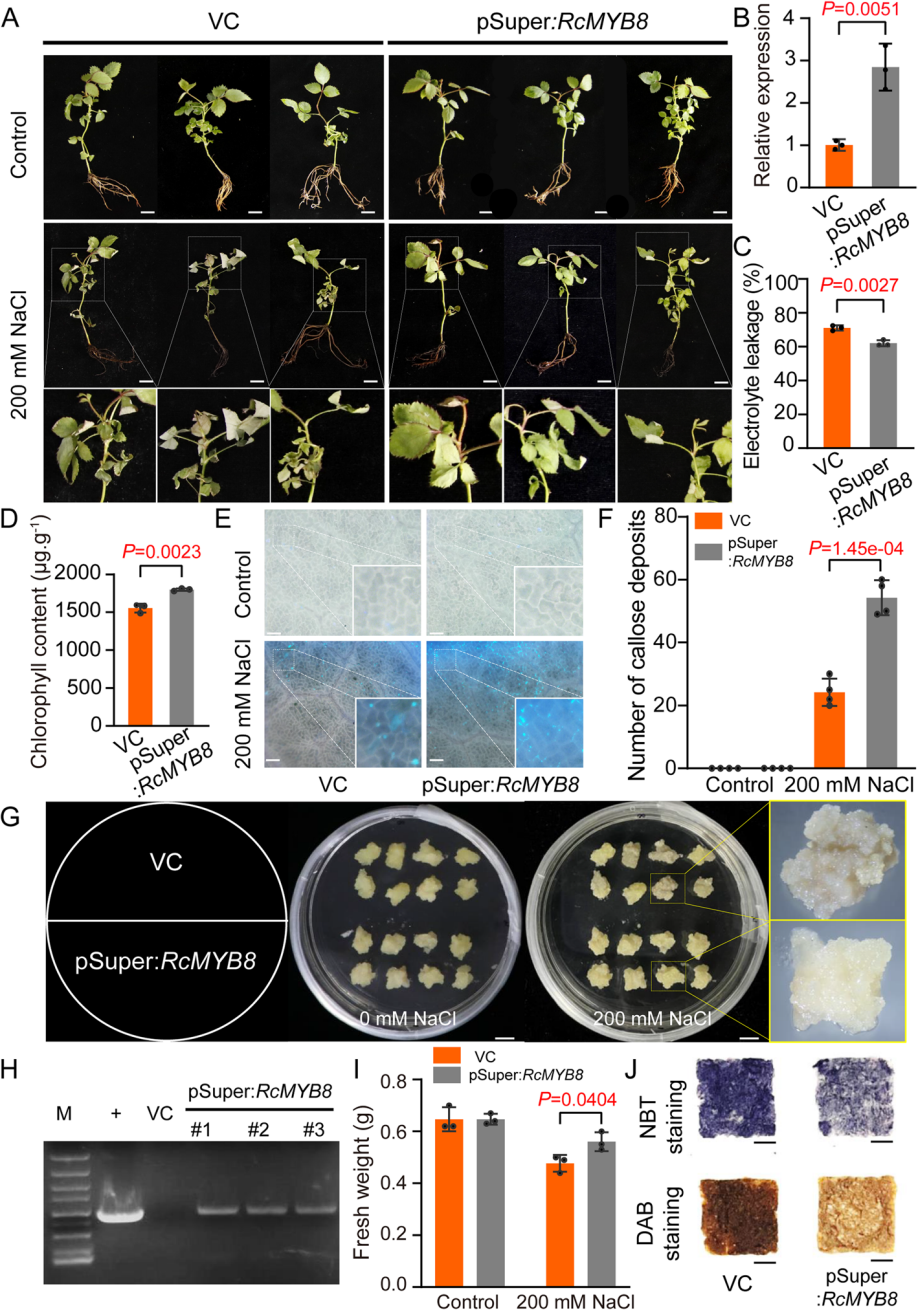
Overexpression of *RcMYB8* improved tolerance to salinity stress. **A** Phenotypes of VC and pSuper:*RcMYB8* under salinity stress. Performance of VC and pSuper:*RcMYB8* rose seedlings under normal and salinity stress conditions. Nine-week-old rose plants were subjected to 0 and 200 mM NaCl for 3 d. Scale bar: 1 cm. **B** Relative expression of *RcMYB8* in VC and pSuper:*RcMYB8*. *RcUBI2* was used as an internal control of three biological replicates. **C** and **D** Electrolyte leakage (**C**) and chlorophyll content (**D)** of leaves in VC and pSuper:*RcMYB8* under salinity stress. **E** and **F** The epidermis of VC and pSuper:*RcMYB8* leaves under a fluorescence microscope, with blue–white spots representing the callosum. Scale bar: 100 μm. **G** Phenotypes of VC and pSuper:*RcMYB8* rose calli treated with 200 mM NaCl for 15 days. Scale bar: 1 cm. **H** PCR analysis of pSuper:*RcMYB8* lines using genomic DNA. +, positive control using the plasmid as template; VC: empty vector transformed plant used as negative control; #1, #2, and #3, pSuper:*RcMYB8* lines. M: 2 kb DNA ladder. **I** Fresh weight in VC and pSuper:*RcMYB8* under salinity stress. **J** NBT and DAB staining of callus tissue after salt stress treatment. Scale bar: 1 cm. Data represent the mean ± SD (*n* = 3)

Next, upon salinity challenge, *RcMYB8* was transiently overexpressed in rose seedlings under a constitutive Superpromoter (VC), thereby deriving *RcMYB8*-overexpressing (pSuper:*RcMYB8*) plants. Compared to the VC control, the pSuper:*RcMYB8* plants exhibited 2.8-fold higher *RcMYB8* level (Fig. 3B). In the 0 mM NaCl scenario, phenotypic differences were absent between VC and pSuper:*RcMYB8* plants. Contrastively, following 3 days of 200 mM NaCl treatment, pSuper:*RcMYB8* plants exhibited fewer wilting phenotypes and a smaller degree of root blackening (Fig. 3A). In addition, VC accumulated higher ion leakage (Fig. 3C) and lower Chl content compared to pSuper:*RcMYB8* plants (Fig. 3D). More callose accumulation in pSuper:*RcMYB8* was also noted compared to the VC control (Fig. 3E, F), indicating that *RcMYB8* may influence the deposition of callose.

We next overexpressed *RcMYB8* in rose calli under normal and 200 mM NaCl, respectively (Fig. 3G). We obtained the pSuper:*RcMYB8* through genomic PCR analysis using the GFP and *RcMYB8* specific primers (Fig. 3H, Supplemental Table S1). Further Quantitative real-time polymerase chain reaction (qRT-PCR) analysis revealed *RcMYB8* was upregulated 2.9-fold in pSuper:*RcMYB8* plants (Fig. S3). Under 200 mM NaCl for 15 d, the VC control callus exhibited severe blackening compared to pSuper:*RcMYB8* callus (Fig. 3G), and the pSuper:*RcMYB8* calli exhibited significantly higher fresh weight (Fig. 3I). The results of NBT and DAB staining showed less concentration of blue and brown colors in pSuper:*RcMYB8* than in VC (Fig. 3J). As implied by these findings, the *RcMYB8* overexpression leads to improved tolerance against salinity by regulating ROS homeostasis.

### Suppression of *RcMYB8* in rose decreases tolerance to drought

As *RcMYB8* exhibited drought-induced expression, we next clarified its role in response to the drought challenge. Firstly, *RcMYB8* in rose leaves was silenced using the VIGS approach under dehydration (0, 6, 12, and 24 h) and rehydration (3 h) conditions (Fig. S4B). After dehydration for 24 h and rehydration for 3 h, TRV-*RcMYB8* exhibited a greater degree of curling than the control group (Fig. S4A). TRV-*RcMYB8* contained significantly less Chl (1319 μg·g^−1^) compared to the TRV control (1551 μg·g^−1^) (Fig. S4C). It is clear from Fig. S4D that compared with TRV controls, the relative fresh weight in TRV-*RcMYB8* was significantly lower at 6, 24 h of dehydration and 6 h of rehydration. Moreover, TRV-*RcMYB8* displayed prominently higher electrolyte leakage in contrast to TRV (Fig. S4F), implying that cell membrane leakage occurred quickly when *RcMYB8* was silenced. Staining with NBT and DAB revealed that TRV-*RcMYB8* accumulated more blue and brown color than TRV (Fig. S4E), respectively, suggesting that *RcMYB8* influenced ROS levels when challenged by drought.

**Fig. 4:**
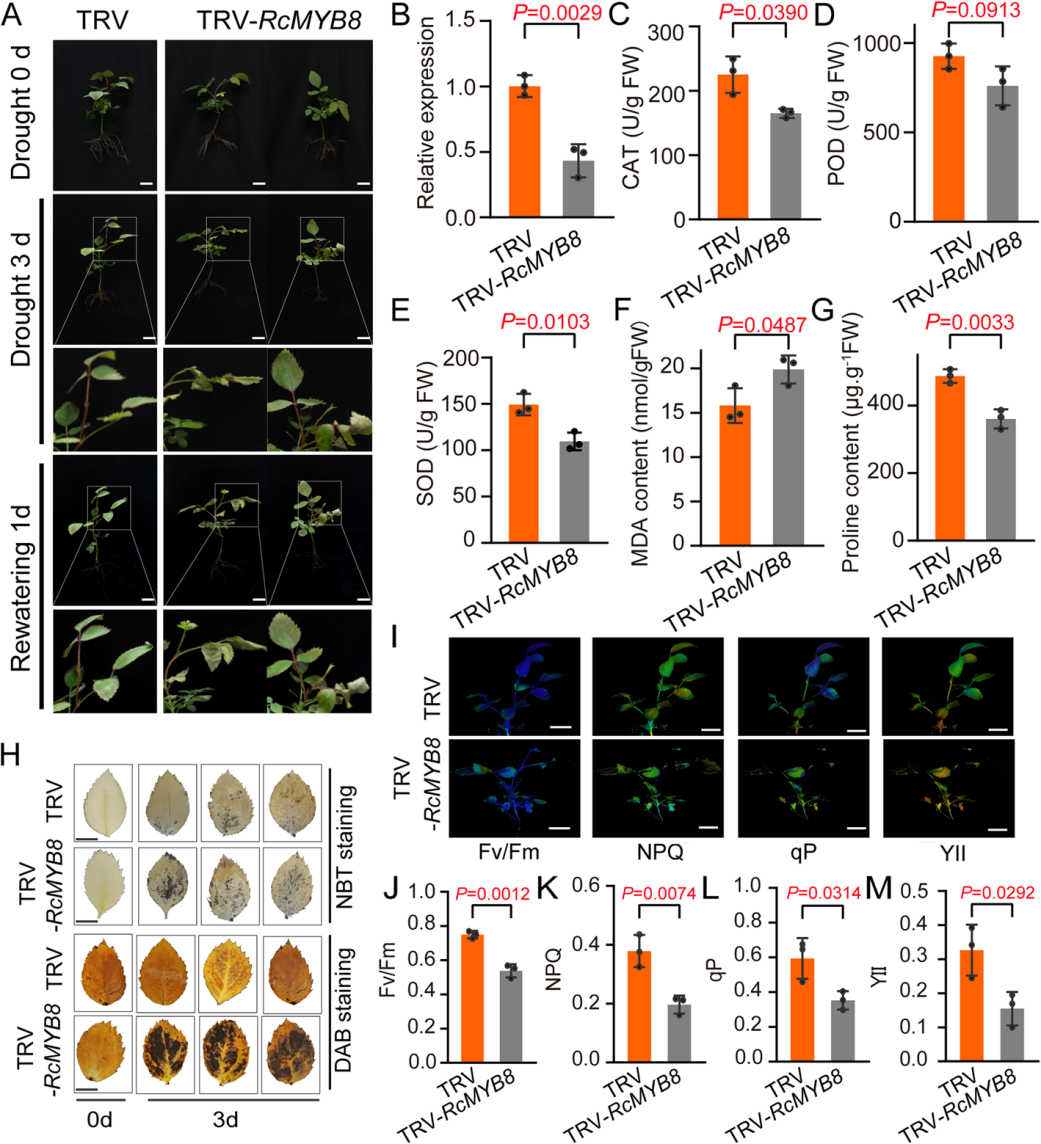
Decreased drought resistance of the *RcMYB8*-silenced rose plants. **A** Phenotype of *RcMYB8*-silenced rose seedlings under drought stress. Three compound leaf stage plants were grown with 20% PEG6000 for 3 d, followed by rewatering for 1 d. Scale bar: 1 cm. **B** Relative expression of *RcMYB8* in TRV and TRV-*RcMYB8*. *RcUBI2* was used as an internal control of three biological replicates

To better examine the role of *RcMYB8* upon drought challenge, we silenced *RcMYB8* in rose seedlings and subjected them to 0-d and 3-d drought stress (20% polyethylene glycol-6000 (PEG6000)), followed by a 1-d rewatering (Fig. 4A). The expression of *RcMYB8* in TRV-*RcMYB8* was only 0.43-fold of that in the TRV control (Fig. 4B). In the normal growth setting, the differences between TRV and TRV-*RcMYB8* were insignificant, while TRV-*RcMYB8* showed more wilting and curling leaves after drought for 3 d (Fig. 4A). Compared to the TRV control, TRV-*RcMYB8* displayed significantly lower enzymatic activities of CAT, POD and SOD (29.8, 17.95 and 26.7%, respectively) (Fig. 4C, D and E), whereas a pronouncedly higher content of MDA, with a value of 19.89 nmol·g^−1^FW (Fig. 4F). Moreover, compared to that in the control, the proline content in TRV-*RcMYB8* was only 360.6 μg·g^−1^FW, showing a markedly lower value compared to the TRV control (487 μg·g^−1^FW) (Fig. 4G). Staining with NBT and DAB revealed that TRV-*RcMYB8* accumulated more blue and brown color than TRV (Fig. 4H).

We also examined the changes in photosynthesis capacity between TRV and TRV-*RcMYB8* after rewatering for 1 d. The photochemical indices, including Fv/Fm, NPQ, qP, and YII, were all significantly higher in the TRV control than in TRV-*RcMYB8*, suggesting that silencing *RcMYB8* resulted in a weaker photosynthesis capacity (Fig. 4I to M). These results clearly suggest that silencing *RcMYB8* decreased tolerance to drought by reducing the proline content, increasing the MDA content, and decreasing the CAT, SOD and POD enzyme activities to balance the ROS level, resulting in weaker photosynthesis capacity.

**C** to **G** CAT activity (**C**), POD activity (**D**), SOD activity (**E**), MDA content (**F**) and proline content (G) of leaves in TRV and TRV-*RcMYB8* under drought stress. **H** NBT and DAB staining of TRV and TRV-*RcMYB8* under drought stress. Scale bar: 1 cm. **I** to **M** Chlorophyll imaging analysis of TRV and TRV-*RcMYB8* rose seedlings under drought stress conditions. Fv/Fm (**J**), NPQ (**K**), qP (**L**), and YII (**M**) indicated in H. Scale bar: 1 cm. Data are the mean ± SD (*n* = 3).

### *RcMYB8* overexpression improves drought tolerance

**Fig. 5:**
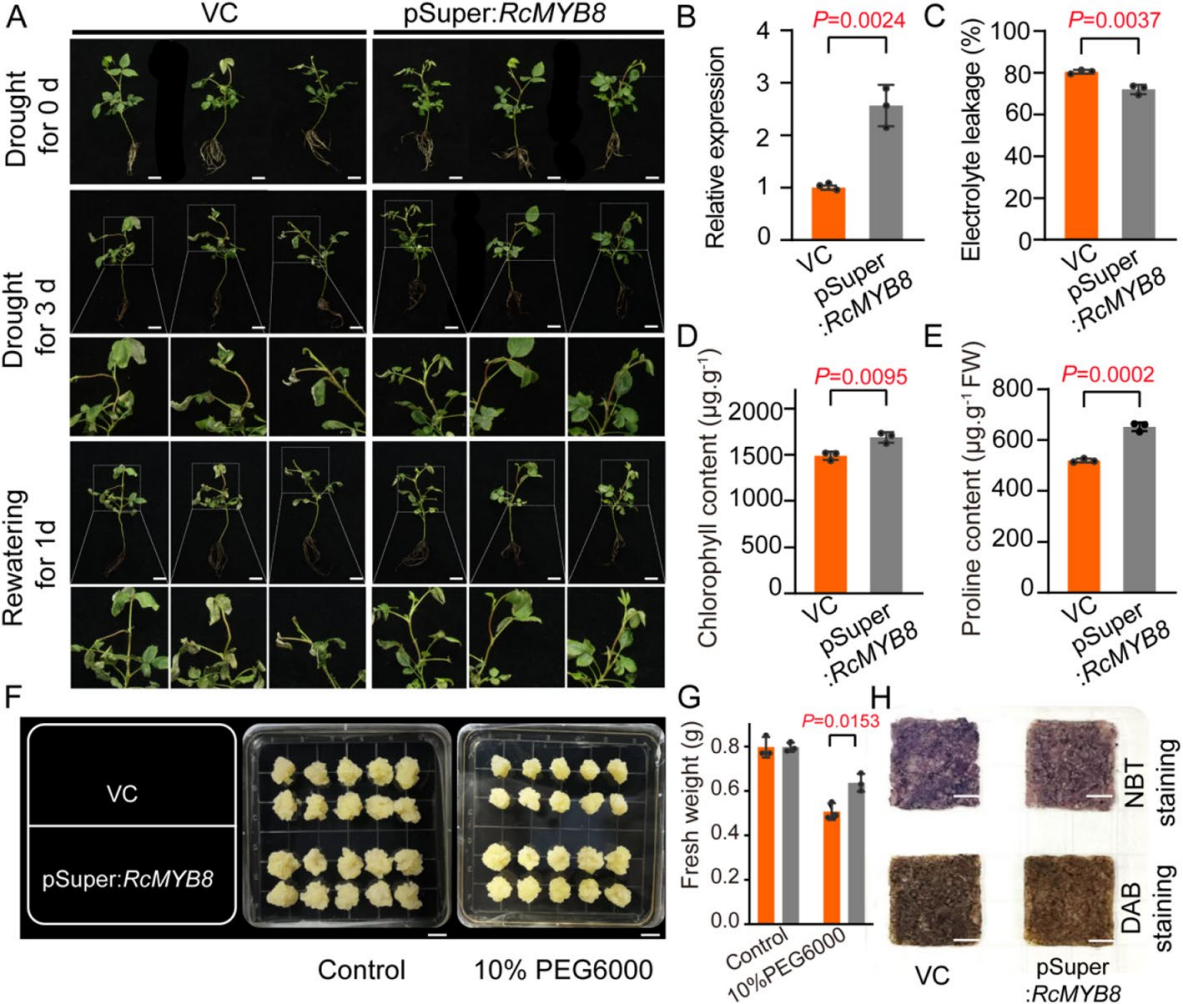
Overexpression of *RcMYB8* improved tolerance to drought stress. **A** Phenotype of VC and pSuper:*RcMYB8* plants under drought stress in rose seedlings. Six compound leaf stage plants were grown with 20% PEG6000 for 3 d, followed by rewatering for 1 d. Scale bar: 1 cm. **B** Relative expression of *RcMYB*8 in VC and pSuper:*RcMYB8*. *RcUBI2* was used as an internal control of three biological replicates. **C** to **E** Electrolyte leakage (**C)**, chlorophyll content (**D)** and proline content (**E**) of leaves in VC and pSuper:*RcMYB8* under drought stress. **F** Phenotypes of VC and pSuper:*RcMYB8* rose calli treated with 10% PEG6000 for 15 days. Scale bar: 1 cm. **G** Fresh weight change in VC and pSuper:*RcMYB8* under drought stress. The error bar indicates the standard deviation (SD) based on three biological repeats. Statistically significant differences were determined by *t* test. **H** NBT and DAB staining of callus tissue after 10% PEG6000 treatment. Scale bar: 1 cm

To probe deeper into the function of *RcMYB8* upon drought challenge, we utilized *RcMYB8* overexpression (pSuper:*RcMYB8*) to study the role of drought challenged rose seedlings. In contrast to the VC control, the pSuper:*RcMYB8* plants exhibited a 2.5-fold higher *RcMYB8* level (Fig. 5B). In normal growth settings, insignificant differences were observed between VC and pSuper:*RcMYB8*, while VC showed more wilting and curling phenotype after drought for 3 d. After rewatering for 1 d, pSuper:*RcMYB8* recovered slightly better than VC (Fig. 5A). We also found that the VC controls showed higher ion leakage (Fig. 5C) and lower Chl content compared with pSuper:*RcMYB8* (Fig. 5D). We also measured the proline content and found that pSuper:*RcMYB8* had a significantly higher proline content than the TRV control (Fig. 5E). In addition, we also overexpressed *RcMYB8* in rose calli in the absence or presence of 10% PEG6000, respectively. The pSuper:*RcMYB8* calli grew much faster than the VC control calli (Fig. 5F). In addition, the pSuper:*RcMYB8* calli exhibited significantly higher fresh weight than that of controls (Fig. 5G). DAB and NBT staining results showed that pSuper:*RcMYB8* accumulated less brown and blue colors than VC (Fig. 5H). These findings illustrate that overexpression of *RcMYB8* enhanced tolerance against drought stress through the ROS scavenging facilitation.

### Levels of *RcPR5/1* and *RcP5CS1* were influenced in TRV-*RcMYB8*

To identify the downstream genes of *RcMYB8*, we firstly identified the genes related to the physiological indicators we tested in *RcMYB8*-silenced and *RcMYB8*-overexpressing plants. We performed a BLAST search on Rosaceae genome database (https://www.rosaceae.org/), where the amino acid sequences of related genes in *Arabidopsis* and rice served as a query. *RcMYB8* improved drought and salt tolerance by influencing ROS dynamic changes and proline accumulation. In addition, previous studies have shown a connection between *RcMYB8* and *RcPR5/1* (Su et al. 2021), and *RcPR5/1* was proved to enhance salinity resistance in rose (Su et al. 2023). We next examined the relevant gene expression corresponding to ROS scavenging, proline synthase and pathogenesis-related-5 (PR5) in both *RcMYB8*-silenced and *RcMYB8*-overexpressing plants. We firstly analyzed POD, SOD, CAT, and proline synthesis and PR5 gene expression using public drought (accession number: PRJNA663119) and salt (accession number: PRJNA42884) transcriptomic datasets (Li et al. 2021; Tian et al. 2018). These genes included 83 *RcPOD*s, 15 *RcSOD*s, one *RcCAT*, two *RcP5CS*s, and 27 *RcPR5*s (Fig. S5A and B).

**Fig. 6:**
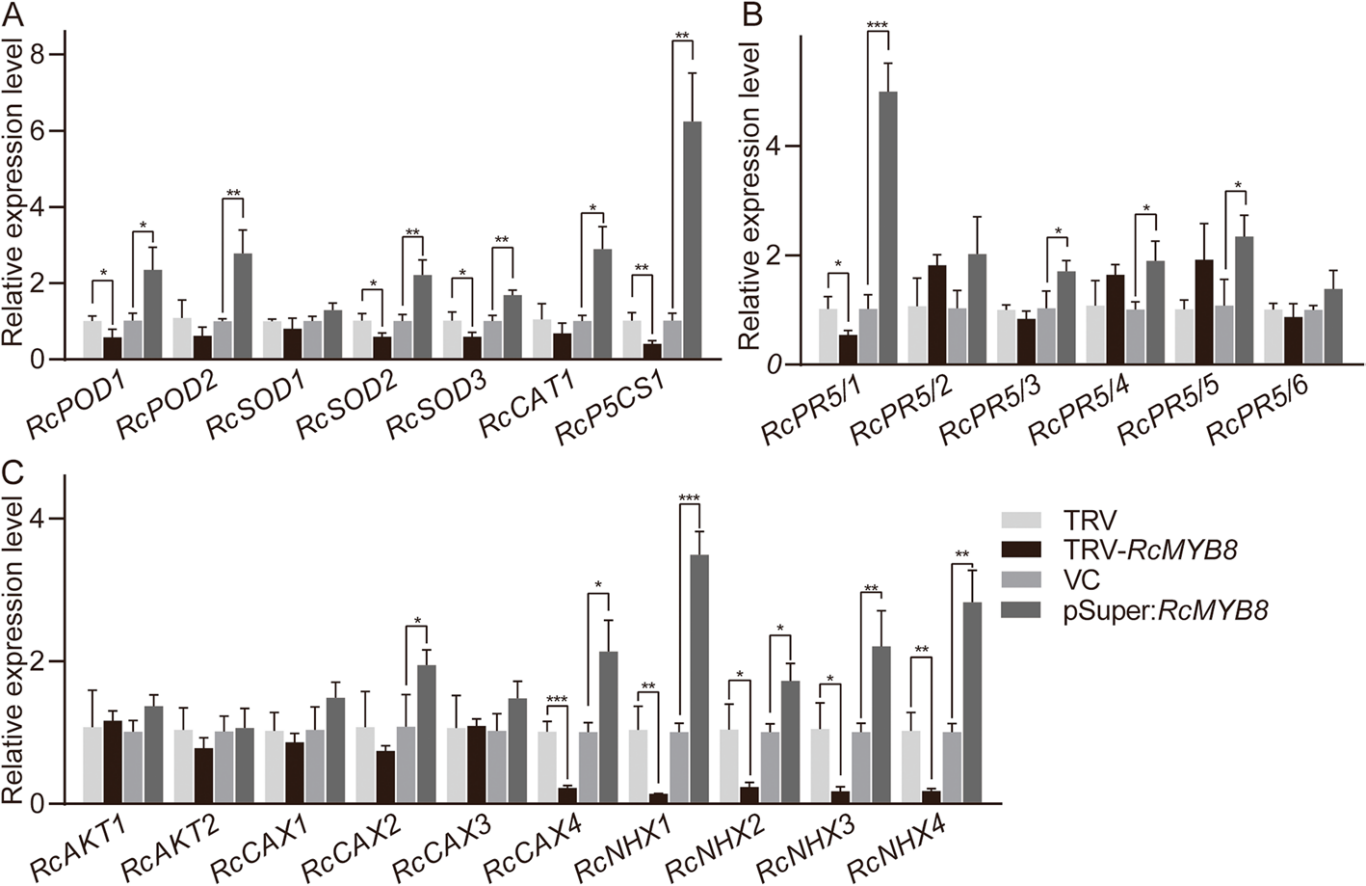
Relative expression changes in oxygen scavenging and proline synthase (**A**), pathogenesis-related-5 (**B**) and ion channel (**C**) genes in *RcMYB8*-silenced and *RcMYB8*-overexpressing rose plants. **A** Relative expression changes of *RcPOD1*, *RcPOD2*, *RcSOD1*, *RcSOD2*, *RcSOD3*, *RcCAT1*, *RcP5CS1* in TRV, TRV-*RcMYB8* and VC, pSuper:*RcMYB8*. **B** Relative expression changes of *RcPR5/1*, *RcPR5/2*, *RcPR5/3*, *RcPR5/4*, *RcPR5/5*, *RcPR5/6* in TRV, TRV-*RcMYB8* and VC, pSuper:*RcMYB8*. **C** Relative expression changes of *RcAKT1*, *RcAKT2*, *RcCAX1*, *RcCAX2*, *RcCAX3*, *RcCAX4*, *RcNHX1*, *RcNHX2*, *RcNHX3*, *RcNHX4* in TRV, TRV-*RcMYB8* and VC, pSuper:*RcMYB8*. *RcUBI2* was used as the control gene. The error bar indicates the standard deviation (SD) based on three biological repeats. Statistically significant differences were determined by *t* test (*, *P* ≤ 0.05; **, *P* ≤ 0.01, ***, *P* ≤ 0.001, ****, *P* ≤ 0.0001)

We then selected the significant drought- or salt-inducible genes to accomplish additional qRT–PCR assessment in TRV-*RcMYB8* and pSuper:*RcMYB8* after drought or salt treatment, respectively (Table. S3). As shown in Fig. 6, four genes (*RcPOD1, RcSOD2, RcSOD3*, and *RcP5CS1*) exhibited significantly depressed gene expression levels, and only *RcP5CS1* was downregulated by more than 50% in TRV-*RcMYB8* and was upregulated by 4.3-fold in pSuper:*RcMYB8* compared with controls (Fig. 6A). For the expression of six *PR5* genes, only *RcPR5/1* was discovered to display a nearly 50% downregulation in TRV-*RcMYB8*, as well as a prominent 5-fold upregulation in pSuper:*RcMYB8* (Fig. 6B). In addition, under salt stress, we found that the K^+^ and Ca^2+^ contents in TRV and TRV-*RcMYB8* did not change greatly, although TRV-*RcMYB8* contained significantly more Na^+^ compared to the TRV. We also identified 32 ion-related genes, including ten *RcAKT*s, nine *RcCAX*s, and 13 *RcNHX*s in the transcriptome database (Fig. S5C). We selected significantly drought- or salt-induced genes, and further qRT–PCR analysis was carried out on TRV-*RcMYB8* and pSuper:*RcMYB8* after salt treatment. Four Na^+^ channel protein genes were significantly downregulated in TRV-*RcMYB8* and upregulated to varying degrees in pSuper:*RcMYB8*. However, the K^+^ channel protein gene did not change significantly in either TRV-*RcMYB8* or pSuper:*RcMYB8*, respectively. Of the four Ca^2+^ channel protein genes (*RcCAX1*, *RcCAX2*, *RcCAX3*, *RcCAX4*), only *RcCAX4* was significantly downregulated in TRV-*RcMYB8*, and the other three genes showed no significant change (Fig. 6C). Based on these results, we infer that *RcMYB8* can modulate *RcPR5/1* and *RcP5CS1* positively in rose plants.

### *RcMYB8* directly bound to the promoters of *RcPR5/1* and *RcP5CS1*

**Fig. 7:**
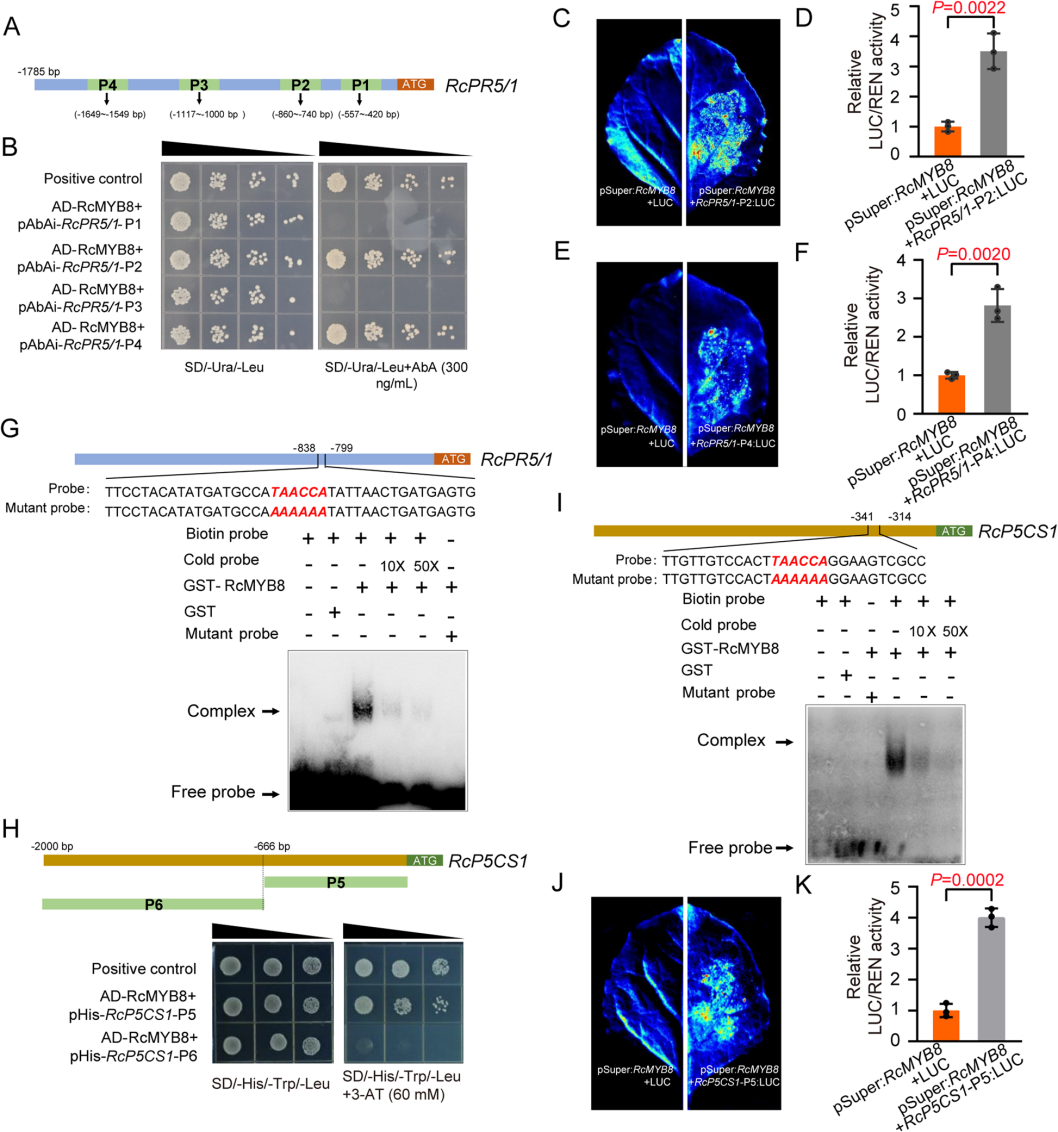
RcMYB8 binds to *RcPR5/1* and *RcP5CS1* in vivo and in vitro. **A** Diagram showing the positions of four fragments containing putative MYB binding sites (P1, P2, P3 and P4) in the 2 kb promoter region of *RcPR5/1*. **B** Y1H analysis showing that RcMYB8 binds to the *RcPR5/1* promoter fragment containing the MYB-binding sites (TAACCA). The promoter of *RcPR5/1* was divided into four fragments. AbA (aureobasidin A), a yeast cell growth inhibitor, was used as a screening marker. The base concentration of AbA was 300 ng/mL. p53 was used as a positive control. **C** to **F** A luciferase complementation imaging assay showing that RcMYB8 accumulates *RcPR5/1*-P2 or *RcPR5/1*-P4 in tobacco leaves. The *Agrobacterium* strain GV3101 (pSoup-p19) harboring different constructs was infiltrated into different regions of tobacco leaves. Luciferase activities were recorded in these regions 3 days after infiltration. **G** EMSA analysis showing that RcMYB8 binds to the TAACCA motif of the *RcPR5/1* promoter. The hot probe was a biotin-labeled fragment of the *RcPR5/1* promoter containing the TAACAA sequence, and the cold probe was a nonlabeled competitive probe (10- and 50-fold larger amounts than that of the hot probe). GST-tagged RcMYB8 was purified. “-” represents the absence and “+” represents the presence of components in the reaction. **H** Binding of RcMYB8 to the *RcP5CS1* promoter in the Y1H assay. The constructs pHis-*RcP5CS1*-P5 and pHis-*RcP5CS1*-P6. **I** Binding of RcMYB8 to the *RcP5CS1* promoter region. A GST-RcMYB8 fusion protein expressed in *Escherichia coli* was purified. The bottom represents the schematic representation of the *RcP5CS1* promoter, and it binding to the biotin-labeled probe contains TAACCA. **J** and **K** A luciferase complementation imaging assay showing that RcMYB8 accumulates *RcP5CS1*-P5 in tobacco leaves. The *Agrobacterium* strain GV3101 (pSoup-p19) harboring different constructs was infiltrated into different regions of tobacco leaves. Luciferase activities were recorded in these regions 3 days after infiltration

*RcP5CS1* and *RcPR5/1* exhibited significantly downregulated expression patterns in TRV-*RcMYB8* and upregulated expression patterns in pSuper:*RcMYB8*. We hypothesize that these two genes might be the downstream genes of *RcMYB8*. Couples of MYB binding sites were evenly distributed in the 2 kb *RcPR5/1* promoter regions (Fig. S6). We then selected four fragments (P1, P2, P3, and P4) that contained different numbers of MYB CREs for Y1H analysis. In yeast cells, 300 ng/mL Aureobasidin A (AbA) suppressed the RcPR5/1 self-activation (Fig. S7). All yeast cotransformed strains with RcMYB8 + *RcPR5/1*-P1, RcMYB8 + *RcPR5/1*-P2, RcMYB8 + *RcPR5/1*-P3, RcMYB8 + *RcPR5/1*-P4 fragments displayed prefereable growth in SD medium deficient in leucine (Leu) and uracil (Ura), but after 300 ng/mL AbA was added to the culture medium. Only the cotransformed RcMYB8 + *RcPR5/1*-P2 and RcMYB8 + *RcPR5/1*-P4 showed normal growth (Fig. 7B), implying that RcMYB8 can bind to the promoter of *RcPR5/1*.

We then used firefly luciferase reporter gene (LUC) analysis to test the binding ability with RcMYB8. The *RcPR5/1*-P2 and *RcPR5/1*-P4 promoter fragments were fused to LUC, and subsequently transformed into *Agrobacterium* GV3101 (Fig. S8), followed by cotransformation of pSuper:*RcMYB8* with *RcPR5/1*-P2-LUC or *RcPR5/1*-P4-LUC in tobacco, respectively (Fig. 7C, E). When pSuper:*RcMYB8* was cotransformed with *RcPR5/1*-P2-LUC or *RcPR5/1*-P4-LUC, RcMYB8 distinctly accumulated relative LUC/REN activity (Fig. 7D, F). To verify the interplay, the full-length *RcMYB8* protein was purified and an electrophoretic mobility shift assay (EMSA) was undertaken, where the labeled probe adopted was biotin-labeled *RcPR5/1* promoter encompassing the sequence of TAACCA. RcMYB8 bound to the *RcPR5/1* promoter (Lane 3, Fig. 7G) and, after addition of competition, the binding ability exhibited a decrease. Additionally, when the binding *cis*-element TAACCA mutated to AAAAAA, no competition was detected with the addition of mutation probes, confirming the specific binding of the sequence TAACCA. The foregoing results suggest binding of RcMYB8 to the promoter of *RcPR5/1*.

There are two proline synthesis genes (*RcP5CS1* and *RcP5CS2*) in rose (Fig. S9), and they exhibited differential expression patterns under drought stress (Fig. S5A). qRT–PCR analysis revealed that *RcP5CS1* exhibited an induced expression pattern under drought for 24 h and 48 h (Fig. S10). We then selected *RcP5CS1* for further binding analysis. Two putative MYB binding sites (*RcP5CS1*-P5 and *RcP5CS1*-P6) were found in its 2 kb promoter regions (Fig. S11). Then, we carried out a self-activation experiment on *RcP5CS1,* and 3-Amino-1,2,4-triazole (3-AT; 60 mM) suppressed the *RcP5CS1* self-activation in yeast cells (Fig. S12). Two yeast strains with *RcMYB8 + RcP5CS1*-P5 and *RcMYB8 + RcP5CS1*-P6 fragments exhibited preferable growth in SD medium deficient in His, Trp and Leu. However, after 60 mM 3-AT was added to the culture medium, only the cotransformed *RcMYB8 + RcP5CS1*-P5 showed normal growth (Fig. 7H). We then performed an EMSA to determine whether RcMYB8 binds directly to the *RcP5CS1* promoter. A 46 bp biotin-labeled DNA fragment in the *RcP5CS1*-P5 regions, which contains the MYB CREs (TAACCA), was used as a probe. When labeled probes were preincubated with GST-RcMYB8 protein, a shifted band was noticed. Contrastively, the GST-RcMYB8 protein binding to the promoter was abolished by adding excess unlabeled probe. Moreover, mutation of the core sequences abolished the binding activity (Fig. 7I). Next we used firefly luciferase reporter gene (LUC) analysis to test the binding ability with RcMYB8. The *RcP5CS1*-P5 promoter fragments were fused to LUC, and subsequently transformed into *Agrobacterium* strain GV3101 (Fig. S13), followed by cotransformation of pSuper:*RcMYB8* with *RcP5CS1-*P5:LUC in tobacco (Fig. 7J). When pSuper:*RcMYB8* was cotransformed with *RcP5CS1*-P5:LUC, RcMYB8 distinctly accumulated relative LUC/REN activity (Fig. 7K). These results suggest that RcMYB8 physically interacts with *RcPR5/1* and *RcP5CS1*.

### Suppression of *RcP5CS1* decreases tolerance to drought

**Fig. 8:**
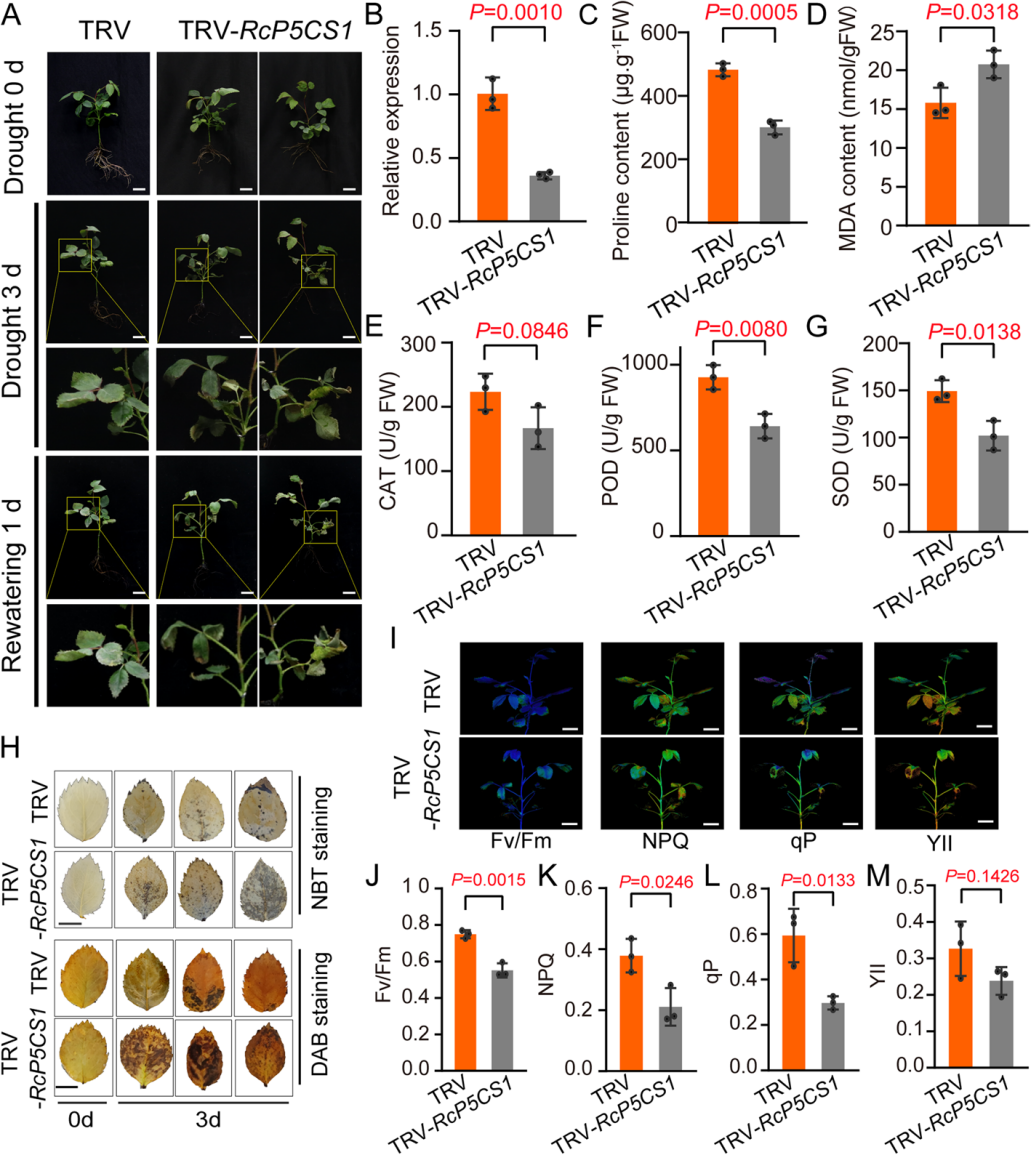
Decreased drought resistance of *RcP5CS1*-silenced rose plants. **A** Phenotype of *RcP5CS1*-silenced plants under drought stress in rose. Two compound leaf stage plants were grown without water supply for 3 d, followed by rewatering for 1 d. Scale bar: 1 cm. **B** Relative expression of *RcP5CS1* in TRV and TRV-*RcP5CS1*. *RcUBI2* was used as an internal control of three biological replicates. **C** to **G** proline content (**C**), MDA (**D**), CAT (**E**), POD (**F**) and SOD (**G**) activity of leaves in TRV and TRV-*RcP5CS1* under drought stress. Data represent the mean ± SD (*n* = 3). **H** NBT and DAB staining of TRV and TRV-*RcP5CS1* under drought stress. Scale bar: 1 cm. **I** to **M** Chlorophyll imaging analysis of TRV and TRV-*RcP5CS1* rose seedlings under drought stress conditions. Fv/Fm (**J**), NPQ (**K**), qP (**L**), and YII (**M**) indicated in I. Scale bar: 1 cm. Data represent the mean ± SD (*n* = 3)

To determine the role of *RcP5CS1* under drought stress, the VIGS method was used to silence *RcP5CS1* in rose seedlings (Fig. 8). After 3 days of drought, TRV-*RcP5CS1* showed more obvious damage in terms of leaf curl and water loss, especially in the tender leaves, which were withered and brittle (Fig. 8A). qRT-PCR analysis showed *RcP5CS1* in TRV-*RcP5CS1* was only 0.36-fold of that in the TRV control (Fig. 8B). We also measured the contents of proline, MDA, CAT, POD, and SOD in both TRV and TRV-*RcP5CS1* (Fig. 8C, D, E, F and G). Compared to the TRV control, the CAT, POD and SOD enzyme activities in TRV-*RcP5CS1* were markedly lower (suppressed by 25.3, 30.7, and 31.6%, respectively). However, the MDA content in TRV-*RcP5CS1* was 20.75 nmol·g^−1^, which prominently surpassed the TRV control value. Compared to the control, the proline content in TRV-*RcP5CS1* was only 305.3 μg·g^−1^ FW, showing a markedly lower value compared to the TRV control (Fig. 8C). Staining with NBT and DAB revealed that TRV-*RcP5CS1* accumulated more blue and brown color than TRV (Fig. 8H).

Furthermore, we examined the changes in photosynthesis capacity between TRV and TRV-*RcP5CS1* after rewatering for 1 d. The photochemical indices, including Fv/Fm, NPQ, qP, and YII, were all distinctly lower in TRV-*RcP5CS1* compared to the TRV control, suggesting that silenced *RcP5CS1* resulted in a weaker photosynthesis capacity (Fig. 8I to M). These results revealed that silencing *RcP5CS1* in rose decreased tolerance to drought.

## Discussion

As a dominant class of plant TFs, MYB plays important roles upon abiotic challenges (Wang et al. 2021). There are four subgroups of MYBs, namely 1R-MYB, 2R-MYB, 3R-MYB, as well as 4R-MYB. Different subgroup members exert diverse functions during the growth and development of plants (Dubos et al. 2010). However, increasing evidence has shown that MYBs belonging to different subgroups are also functionally consistent, and the same subgroup gene also plays different roles in different plants. For example, *Arabidopsis* 3R-MYB *AtMYB3R-1* and *AtMYB3R-4* are demonstrated to exert a function in cytokinesis, while in rice, the same subgroup member *OsMYB3R-2* can improve tolerance against abiotic challenges (Haga et al. 2007; Dai et al. 2007). Two 1R-MYB-type genes, *MCB1* and *MCB2,* in barley participate in the expression of light and circadian clock regulation in leaves (Churin et al. 2003), while the same group member, *GmMYB176,* in soybean regulates isoflavone biosynthesis (Yi et al. 2010). Although MYBs have been extensively studied in different plant species, there are few basic reports of this kind in roses. Our study first identified the rose 2R-MYB gene *RcMYB8*, which has high homology with *PtMYB134* (Mellway et al. 2009) and *AtMYB96* (Seo et al. 2011). The expression results showed that drought or salinity can rapidly induce the expression of *RcMYB8* (Fig. 1B, C), indicating a probably role of *RcMYB8* in drought and salinity challenges. Besides, we found that *RcMYB8* was closely related to proanthocyanidin and anthocyanin synthesis genes during the construction of the evolutionary tree (Fig. 1A), implying that *RcMYB8* might also play a role in anthocyanin synthesis.

Under drought or salinity stresses, plants accumulate some physiological indicators [relative conductivity (REL), Chl content, chlorophyll fluorescence, callose accumulation] to improve their adaptability under these unfavorable conditions (James et al. 2018; Tang et al. 2019; Su et al. 2023). Among them, REL is a vital indicator reflecting the severity of cellular membrane impairment (James et al. 2018). Silenced *RcMYB8* in rose leaves resulted in higher REL than in the leaves of the control (Fig. S2C, S4F), Overexpression of *RcMYB8* results in lower REL compared to the control (Fig. 3C, 5C), indicating a potential role of *RcMYB8* in maintaining the integrity of plant cell membrane. When plants are challenged by drought, due to the water content reduction in leaves, the rate of chlorophyll synthesis is slow, leading to an increased rate of chlorophyll degradation (Liu et al. 2018). Therefore, Chl content is another important factor in determining plant responses to adverse stress conditions. Under drought or salt stresses, silencing *RcMYB8* resulted in lower Chl contents than those in controls (Fig. S2D, S4C), Overexpression of *RcMYB8* results in higher chlorophyll content compared to the VC (Fig. 3D, 5D), implying that suppression of *RcMYB8* resulted in more damage to the leaves.

Chlorophyll fluorescence analysis can be used to quantify the absorption of optical energy directly used for photochemistry, and to estimate photosynthetic performance in the biotic or abiotic stress settings (Moustakas et al. 2021). The effective quantum yield (YII) and photochemical quenching coefficient (qP) of plant photochemical energy conversion were significantly reduced after short-term drought stress, and the nonphotochemical quenching coefficient (NPQ) along with Fv/Fm (maximum quantum yield of PSII) were significantly reduced under long-term drought stress (Shin et al. 2021). In our study, *RcMYB8*-silenced plants exhibited photoinhibition and damage to the photosynthetic apparatus (Fig. 4J, K, L and M), implying that the light and energy conversion of silenced *RcMYB8* plants was weakened, which repressed the drought resistance of plants. Previous research has shown that there exists a connection between photosynthesis and proline metabolism (Szabados and Savouré 2010), implying that proline accelerates rose photosynthesis and stabilizes cellular homeostasis under drought stress.

For plants, ion stress is one of the main consequences of salt toxicity (Liu et al. 2022b), in which the Na^+^ deposition and K^+^ level instability constitute one of the ways of causing ion stress. Therefore, keeping the Na^+^/K^+^ ratio equilibrium has become a critical mechanism to cope with salinity challenge (Brindha et al. 2021). Overexpression of tomato *SlMYB102* resulted in less Na^+^ and more K^+^, thus keeping the ratio of Na^+^/K^+^ stable (Zhang et al. 2020). Silencing *RcbHLH59* in rose resulted in an unbalanced Na^+^/K^+^ ratio with changes in Na^+^ (Su et al. 2023). In addition to Na^+^ and K^+^, increasing evidence has shown that Ca^2+^ also plays a unified and coordinated role in salinity stress at the cellular and tissue levels, functioning to translate external signals into intracellular responses (Köster et al. 2019). In our study, no significant differences in Ca^2+^ and K^+^ were found between TRV and TRV-*RcMYB8* (Fig. 2C, D), while Na^+^ exhibited higher levels in TRV-*RcMYB8* than in TRV controls (Fig. 2E). *RcMYB8* mainly affects the Na^+^/K^+^ ratio through the change in Na^+^ content (Fig. 2F), resulting in an imbalanced Na^+^/K^+^ ratio and influencing Na^+^ transporters on both the plasma membrane and the mitochondria to mediate Na^+^ signaling. Interestingly, insignificant differences were noted between TRV and TRV-*RcMYB8* regarding Ca^2+^ content, while *RcCAX4* was significantly reduced in TRV-*RcMYB8* (Fig. 6C), which may be due to a series of complex reactions occurring when faced with stress. These results revealed a coordination relationship among different ions, and the pivotal transporters need to be further investigated.

ROS are highly reactive molecules that are produced when plants suffer from drought or salinity stress. ROS can damage the cell membranes, reconstructing DNA and proteins, leading to lipid peroxidation and deposition of MDA (Mellacheruvu et al. 2016; Tang et al. 2019). Plants accumulated more ROS have a negative influence on their growth, development, and response to environmental stress. Under drought stress, plants experience water deficiency, resulting in the ROS deposition and oxidative stress. Moreover, intricate mechanisms have been evolved by plants for scavenging higher levels of ROS, including the production of antioxidant enzymes like CAT, SOD and POD. These enzymes can convert excessive and harmful active oxygen into harmless water (Noctor and Foyer 1998; Xu et al. 2016). Latest works have illustrated the crucial role of ROS level regulation for plant responses to drought stress. When subjected to drought stress, *CaCIPK3*-overexpressing plants have a stronger scavenging ability of ROS (Ma et al. 2021). In our current work, under drought stress, the CAT, SOD and POD activities of *RcMYB8*-silenced plants decreased (Fig. 4), while the contents of H_2_O_2_ and O_2_^−^ were increased by DAB and NBT staining (Fig. 3-5), indicating that more damage was caused when *RcMYB8* was repressed in rose plants.

Proline not only exerts a cytoprotective function against abiotic challenge as a stabilizer (Ben et al. 2012), but also plays an active role in osmoregulation, protecting enzyme structure, stabilizing membrane, and defending hydroxyl free radicals. Therefore, the proline content can be considered a strength of stress tolerance. As a molecular partner, proline has been shown to be involved in the protection of protein integrity and the enhancement of different key enzyme activities (Ashraf and Foolad 2007; Szabados and Savouré 2010). Many studies have shown that there are merely two *P5CS* genes in most plant species, which are key biosynthetic enzymes for proline (Strizhov et al. 1997; Fujita et al. 1998; Ginzberg et al. 1998). These two genes are usually induced by different regulatory mechanisms and play different roles. For example, one *P5CS* gene functions during the development of plant embryos and seedling growth, while the other functions to cope with environmental stress (Székely et al. 2008). In our study, we also found that there are two *RcP5CS* genes in rose. Transcriptome data analysis showed that *RcP5CS1* exhibited a drought-induced expression pattern compared with *RcP5CS2*, implying that *RcP5CS1* might have vertical functions in responses to abiotic stresses. The proline contents in both TRV-*RcMYB8* and TRV-*RcP5CS1* were lower than those in the controls (Fig. 4G, 8C), indicating that *RcMYB8* could regulate the expression of *RcP5CS1*. In addition, pairs of MYB binding sites were unevenly distributed in the promoter regions of *RcP5CS1*. Moreover, Y1H, LUC and EMSA experiments showed that RcMYB8 can directly target the *PcP5CS1* promoter in vitro and in vivo. These results revealed that *RcMYB8* enhanced tolerance to salinity or drought stress by regulating *RcP5CS1*.

As a β-1,3-glucan polysaccharide having β-1,6 branches, callose is implicated in many processes of plant development, and responses to abiotic and biotic challenges (Wu et al. 2018). Previous studies revealed that RcbHLH59 interacts with RcPR5/1 and positively regulates salinity stress tolerance by influencing callose deposition in rose plants (Su et al. 2023). In addition, RcPR5/1 interact with RcPR4/1, and these two proteins belong to the PR protein family, which is known to play a role in plant responses to stress (Su et al. 2021). In this study, it was found that when *RcMYB8* was overexpressed, the content of callose rose in contrast to that in the control (Fig. 3E), indicating that *RcMYB8* was involved in the accumulation of callose, thereby enhancing salt tolerance.

Plant species under salinity or drought stress will close their stomata and thus reduce water loss, resulting the same phenotype or symposium. This suggest that there existed a crosstalk or the same possible mechanism that participated in. Our results illustrate that RcMYB8 targets *RcPR5/1* (Fig. 7A-G), influencing callose deposition and the balance of Na^+^/K^+^ (Fig. 2F; Fig. 3E, F), resulting in enhanced tolerance to salinity stress (Fig. 3A, G). Additionally, RcMYB8 can bind to the *RcP5CS1* promoter region (Fig. 7H-K), inducing drought tolerance by upregulating proline content to scavenge ROS (Fig. 4C-G; Fig. 5E, H). Therefore, we predict that there might be a crosstalk between the module of RcMYB8-RcP5CS1 under salt stress and RcMYB8-RcPR5/1 under drought stress. Further evidence or experiments are needed to investigate this.

**Fig. 9:**
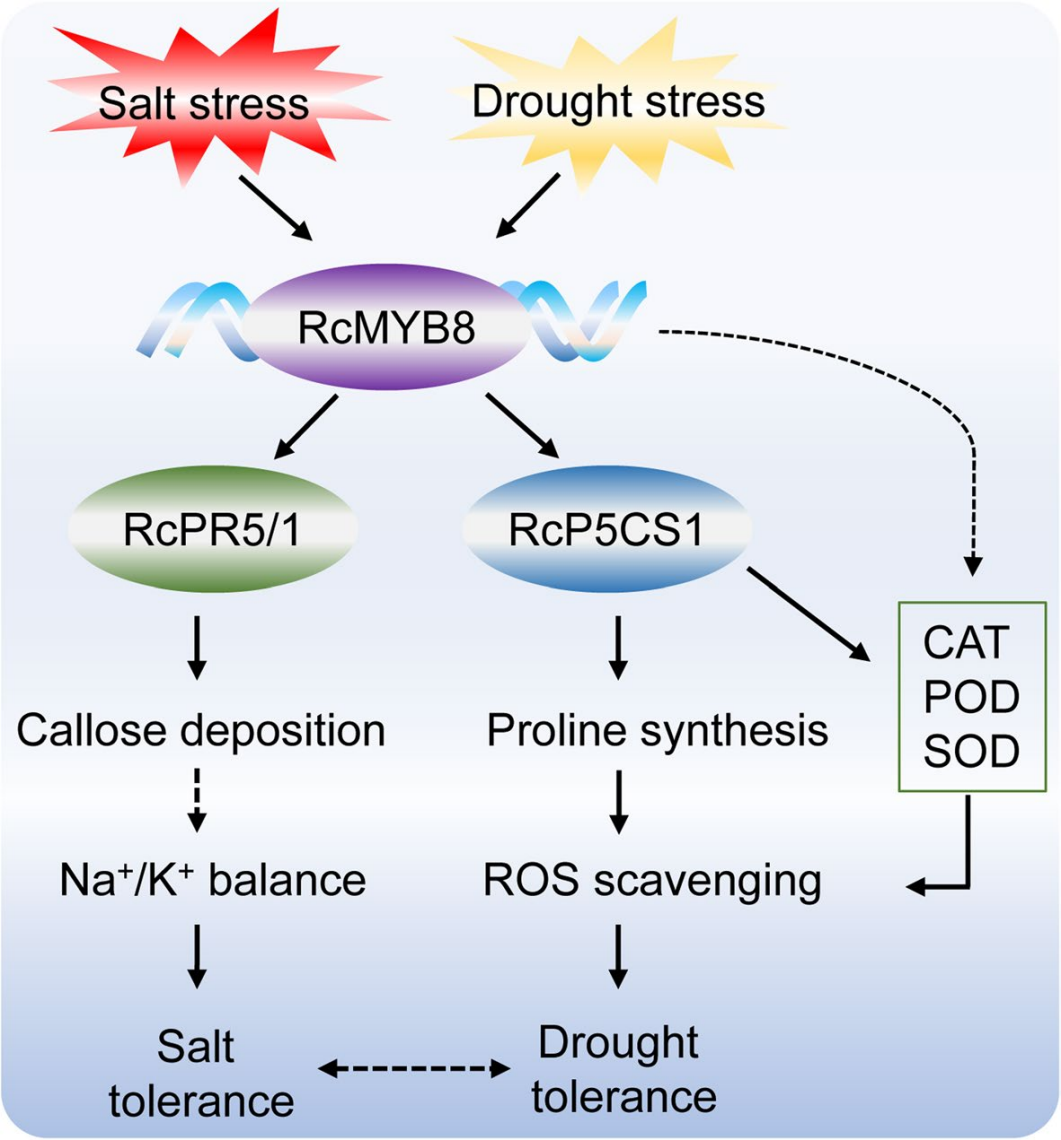
A proposed model of the function of RcMYB8 in the regulation of salt and drought tolerance. Salinity and drought stress induced transcriptional expression of *RcMYB8*. Then, RcMYB8 binds to the promoters of both *RcPR5/1* and *RcP5CS1*, influencing the callose deposition and proline contents. This in turn promotes Na^+^/K^+^ balance and enhances ROS scavenging ability. Thus, tolerance to salinity and tolerance to drought stress are enhanced

In conclusion, we characterized a novel regulatory module that is connected by RcMYB8. RcMYB8 functions as a bridge and regulates *RcPR5/1* and *RcP5CS1* via two unique pathways upon challenge by salinity and drought in rose. The first regulatory module is RcMYB8-RcPR5/1. RcMYB8 targets *RcPR5/1*, influencing callose deposition and the balance of Na^+^/K^+^ and giving enhanced tolerance to salinity stress. The second module is RcMYB8-RcP5CS1. RcMYB8 can bind to the *RcP5CS1* promoter region and induce drought tolerance by upregulating the proline content to scavenge ROS. These two regulatory modules represent the complexity of *RcMYB8* regulatory networks in rose plants (Fig.9).

## Materials and methods

### Plant materials and cultivation settings

The in vitro propagation buds of rose (*Rosa chinensis* ‘Old Blush’) from Qingdao Agricultural University were used as materials, which were grown for 7 weeks on Murashige and Skoog (MS) alkali salts. The cultivation settings were 25 °C, 16-h light/8-h dark photocycle, as well as 50-60% RH. Next step was a 2-week cultivation of rose plants using 1/4 Hoagland solution, followed by treatment of the plants for 0, 3, 6, 12, 24 and 48 h using NaCl (200 mM) or PEG6000 (20%). Utilizing the sterile young rose leaves, callus induction was accomplished on callus induction medium (CIM), while subsequent somatic embryo induction was undertaken on embryos induction medium (EIM) according to Liu et al. (2021). The callus was cultured without light at 26 °C and renewed every 4 weeks. Each experiment consisted of three independent plants.

### Phylogenetic analysis

For sequence analysis, Genedoc was used to perform a visual alignment of the homologous RcMYB8 protein. The phylogenetic tree of 29 MYB proteins with known functions from other plant species (Supplementary Table. S2) were constructed in MEGA X using the neighbor connection (NJ) method (Kumar et al. 2018).

### Quantitative real-time PCR analysis

A FastPure Isolation Kit (Vazyme Biotech, Nanjing, China) was utilized for the total RNA sampling from rose leaves and, subsequently, a HiScript III All-in-One RT SuperMix Perfect for qPCR from the same manufacture was used to reverse transcribe the total RNA, thereby deriving cDNA. qRT–PCR was accomplished following a prior procedure (Su et al. 2023). Gene expression was normalized against the internal control *RcUBI2*, while the level of each gene expressed was computed by 2^-∆∆CT^ approach. Supplementary Table S1 details the adopted qRT–PCR primers.

### Subcellular localization of *RcMYB8*

With the assistance of specific primers (Supplemental Table. S1), *RcMYB8*-GFP was formed by inserting *RcMYB8* coding region between the *Sa*l I and *Hin*d III sites in pCAMBIA 1300 vector. After transforming both RcMYB8-GFP and the vector into *Agrobacterium tumefaciens* CV3101, infection proceeded into tobacco (*Nicotiana benthamiana*) leaves that were aged 4 weeks. Following 2 days of placement away from light, the lower epidermal samples were gathered from tobacco leaves, which were subjected to 4′, 6-diamidine-2′-phenylindole dihydrochloride (DAPI, 10 μg/mL) staining, and finally photographed under a TCS SP8 laser scanning confocal microscope (Leica, Germany).

### Transcriptional activation assay

With the utilization of specific primers (Supplementary Table. S1), *RcMYB8* coding sequence was subjected to PCR amplification, and subsequently connected to pGBKT7 vector encompassing the DNA binding domain of GAL4. After transforming fusion vector pGBKT7-*RcMYB8* (BD-RcMYB8), empty body (BD), as well as positive and negative controls into yeast strain yeast two-hybrid (Y2H) GOLD (Weidi, Shanghai, China), coating proceeded with SD/−Trp and SD/−Trp-His growth medium, respectively. After growing in a constant temperature oven at 28 °C for 3-5 days, the samples were photographed.

### VIGS

The VIGS approach was conducted as previously reported (Su et al. 2023). The 200- and 483-bp fragments in the non-conserved zone of the 3′ region of *RcMYB8* and the 5′ region of *RcP5CS1* were selected as silent fragments. Through their insertion into pTRV2 vector, plasmids pTRV2-*RcMYB8* and pTRV2-*RcP5CS1* were derived, which were transformed subsequently into *Agrobacterium tumefaciens* strain GV3101. After supplementing with AS (40 μM), MES (10 mM), kanamycin (100 mg/L) and rifampicin (100 mg/L), every transformant was cultured in YEB medium. Folling overnight culture (200 rpm) at 28 °C, we collected the cellular strain and centrifuged the cell body (5000×g, 8 min) using suspended bacteria (200 μM AS, each 10 mM MgCl_2_ and MES), followed by rectification of the OD600 to 0.8-1.0. Cells containing pTRV2-*RcMYB8*, pTRV2-*RcP5CS1* or pTRV2 were blended with pTRV1 cells in a 1:1 volume and incubated in darkness for 4 h. Then, the nutrient solution-cultured rose plants were infiltrated with strains containing TRV-*RcMYB8* or TRV-*RcP5CS1* as the TRV plasmid (negative control) under 0.7 atm for 10 min and then circulated twice. After soaking, deionized water was used to wash the plants, in order to eliminate the osmotic buffer. Finally, the plants were cultured in darkness at 8 °C for 3 days and then treated under an aqueous scenario containing NaCl (0 or 200 mM) or PEG6000 (20%) in identical cultivation conditions.

### Transient overexpression and genetic transformation

Transient overexpression of *RcMYB8* was accomplished following a former procedure (Luo et al. 2021). First step was cloning of open reading frame (ORF) of *RcMYB8* into the pCAMBIA1300 vector, thereby yielding *RcMYB8*-overexpressing plants (pSuper:*RcMYB8*). Through centrifugation, *A. tumefaciens* GV3101 carrying pCAMBIA 1300 (VC) or pSuper:*RcMYB8* was gathered, which was resuspended using buffer solutions (200 μM AS, each 10 mM MgCl_2_ and MES), followed by rectification of the OD600 to 0.8 for 3-4 h. Then, hydroponic plantlets with similar growth conditions were selected for infiltration in a vacuum of 0.7 atm pressure. Next, the infiltrated plants were incubated in darkness at 8 °C for 3 days and treated with PEG6000 (20%) or NaCl (0 or 200 mM) in identical growth settings.

Genetic transformation of rose calli was conducted following previous studies procedure with little modification (Liu et al. 2021). The bacterial solution was first cultured overnight on a shaker, gathered through centrifugation and resuspended using buffer solutions (MS medium with 100 μM AS and 60 g/L glucose), and OD600 was adjusted to 0.5. The culture was then incubated at 200 r/min and 28 °C for 2 h. Then, the same growth state of callus was soaked for 15 minutes in the bacterial suspension using a vacuum pump of 0.7 Mpa, and then subjected to a 4-d cocultivation at 22 °C devoid of light using a co-culture medium (CM). For cultivation of somatic embryos, a screening medium involving antibiotics (300 mg/L Cefotaxim and 20 mg/L Hygromycin) was utilized. Through both qRT-PCR and genomic PCR, identification of positive pSuper:*RcMYB8* plants was accomplished with the utilization of specific primers (Supplementary Table S1). The screened pSuper:*RcMYB8* and VC calli were treated on a medium containing 200 mM NaCl or 10% PEG6000, respectively. At least 16 calli were used for each experiment.

### Physiological assay

Chlorophyll quantification was accomplished as per a prior procedure (An et al. 2021). A chlorophyll fluorescence imager (IMAG-MAX/L; WALZ, Germany) was used to measure the image and parameters of qP, NPQ, Fv/Fm, and Y(II), with each 3 technical and biological replicates.

To assess the rate of ion leakage, plant sample was weighed (0.2 g) and placed for 8-12 h in deionized water (10 mL) to measure their conductivity. The mixture was boiled for 30 minutes and then cooled to room temperature, and the conductivity was measured again. The relative electrical conductivity was calculated as the conductivity prior to high boiling as a percentage of post-boiling conductivity.

Diaminobenzidine (DAB) along with nitro blue tetrazolium (NBT) were used to determine the H_2_O_2_ and O_2_^−^ depositions in leaves by histochemical staining. The relative fresh weight is referred to as the indicated timepoint (drought 6, 12, 24, rewatering 3 h) weight versus the weight at 0 h. The enzymatic activity assessment for Peroxidase (POD), Superoxide Dismutase (SOD), Catalase (CAT) and proline was undertaken separately via the corresponding assay kits (Solarbio, China). Thiobarbituric acid method (TBA) was employed for quantifying MDA levels. Three biological replicates were used for each physiological index.

For the contents of Na^+^, K^+^ and Ca^2+^, we firstly dried the salt-stressed sample in an 80 °C dryer, ground and weighed 0.1 g, put it into a digestive tube, added 12 mL of HNO_3_:HCLO_4_ (V:V = 5:1), mixed it with acid, covered it, and let it stand overnight. Acid digestion proceeded on the next day at 160-170 °C on a temperature-controlled digestion instrument, and finally, the sample solution was transferred to a volumetric flask without loss, and deionized water was used to bring the volume to 30 mL. Finally, an optical emission spectrometer (Perkin Elmer, USA) was utilized to assess the Na^+^, K^+^ and Ca^2+^ levels in the liquid, respectively. Each test was conducted using three biological replicates.

### Callose staining

Callose staining was determined as described previously (Su et al. 2023). After treating leaves infected with TRV and TRV-*RcMYB8* using NaCl solution (0 and 200 mM), they were subjected to a 1-h aniline blue solution (Solarbio, Beijing, China) staining devoid of light. Green fluorescent samples were deemed as callose (Luna et al. 2011), which were surveilled using an Axio Scope A1 microscope (Carl Zeiss). With the aid of ImageJ (https://imagej.nih·gov/ij/), the callose quantity was identified.

### Y1H

To produce an effector construct, cloning of *RcMYB8* coding sequence into pGADT7 vector was accomplished. After cloning *RcP5CS1* promoter fragments into pHIS2 vector, they were transferred into Y1H yeast cells with pGADT7-*RcMYB8*. For spot analysis, we chose and grew the transformants on SD/−Trp-His-Leu and SD/−Trp-His-Leu + 3-AT plates. The *RcPR5/1* promoter fragment was cloned into the *Bst*B I-digested pABAi vector and cotransformed into Y1H yeast using pGADT7-*RcMYB8*. We chose and grew the transformed positive yeast colonies on SD/−Ura plates, and subsequently transferred them severally to SD/−Ura/−Leu + AbA and SD/−Ura/−Leu plates for spot analysis.

### Luciferase assay

Cloning of *RcMYB8* CDS as an effector into the pCAMBIA 1300 vector was accomplished. The pGreenII 0800-LUC vector was inserted with *RcPR5/1* promoter sequence (P2, P4) and *RcP5CS1* promoter sequence (P5) as a reporter. After transformation of the plasmid into *Agrobacterium* strain GV3101, cultivation of vector-transformed *A. tumefaciens* proceeded under 200 rpm at 28 °C until OD600 = 1.0. Then, the *Agrobacterium* was suspended in liquid medium (200 μM AS, each 10 mM MgCl_2_ and MES), incubated for 3 h and filtered into the back of the tobacco leaf. After 3 days of growth in the dark, the leaves were sprayed with sodium d-fluorescein (Sangon Biotech, Shanghai, China). A live plant fluorescence detector (SH-Advance 523, Hangzhou) was employed for the fluorescence surveillance, while a dual luciferase reporting reagent (Vazyme Biotech, Nanjing, China) was utilized to evaluate the activity levels of luciferase (LUC) and renal luciferase (REN) in fireflies. Each test was conducted using three biological replicates.

### EMSA

The ORF of *RcMYB8* was inserted into the vector expressing pGEX4T-1, followed by transfer into *E. coli* BL21(DE3) for expression, added 100 mM Isopropyl-β-D-thiogalactopyranoside (IPTG), thereby producing recombinant GST-RcMYB8 protein. A chemiluminescent EMSA kit (Beyotime, Shanghai, China) was used for the binding reaction. The probe and mutant fragments of the *RcPR5/1* and *RcP5CS1* promoters were selected and labeled with biotin, and the same unlabeled fragments were used as competitors. Single-chain 5′ end biotin-labeled probes were synthesized by Shanghai Sangon Biotech (China).

### Statistical analyses

SPSS ver. 25.0 (SPSS Inc., Chicago, IL, USA) was used to undertake the entire statistical analyses. Data were processed by Student’s *t* test or univariate analysis of variance (ANOVA), and subsequently through least significant difference (LSD) approach. Individual experimental statistics are presented as means ± standard deviations (SDs).

### Supplementary Information

**Additional file 1: Supplementary Figure S1**. Domain structure and sequence alignment of RcMYB8 and R2R3 MYB proteins from other species. **Supplementary Figure S2.** Silencing of *RcMYB8* decreased salt tolerance in rose leaves. **Supplementary Figure S3.** Relative expression of *RcMYB8* in VC and pSuper: *RcMYB8*. **Supplementary Figure S4.** Silencing of *RcMYB8* decreased drought tolerance in rose leaves. **Supplementary Figure S5.** Expression patterns of genes  related to ROS scavenging and proline synthesis (A), pathogenesis-related protein 5 (B), and Na^+^, K^+^, Ca^2+^ transporter (C) under drought and salt stress treatments. **Supplementary Figure S6.** Sequence information of the *RcPR5/1* promoter. **Supplementary Figure S7.** Self-activation detection of pAbAi-*RcPR5/1* in yeast. **Supplementary Figure S8.** Schematic of the effector and reporter vector of *RcMYB8* and *RcPR5/1*. **Supplementary Figure S9.** Domain structure and sequence alignment of *RcP5CS1* with other plant P5CS proteins. **Supplementary Figure S10.** Expression profiles of *RcP5CS1* under drought conditions at indicated time points. **Supplementary Figure S11.** Sequence information of the *RcPR5/1* promoter. **Supplementary Figure S12.** Self-activation detection of pHis-*RcP5CS1* in yeast. **Supplementary Fig. S13.** Schematic of the effector and reporter vector of *RcMYB8* and *RcP5CS1*.

**Additional file 2: Supplementary Table S1.** The primers sequences used in this study. **Supplementary Table S2.** List of genes used in phylogenic analysis of RcMYB8. **Supplementary Table S3**. List of accession numbers in this study.

## Data Availability

The authors confirm that all data in this study are included in this published article (and its supplementary information file).

## Abbreviations

AbA: Aureobasidin A
bHLH: basic/helix-loop-helix
bZIP: basic leucine zipper
EMSA: Electrophoretic mobility shift assay
GFP: Green fluorescent protein
IPTG: Isopropyl-β-D-thiogalactopyranoside
LEA: Late embryogenesis abundant
LUC: Firefly luciferase
MYB: Myeloblastosis
MS: Murashige & Skoog medium
NAC: NAM, ATAF1,2, CUC2
ORF: Open reading frame
P5CS: Pyrroline-5-carboxylate synthase
PEG-6000: Polyethylene glycol-6000
PR: pathogenesis related
qRT-PCR: Quantitative real-time polymerase chain reaction
REN: Renilla luciferase
TRV: Tobacco rattle virus
TF: Transcription factor
VIGS: Virus induced gene silencing
Y1H: Yeast one-hybrid
Y2H: Yeast two-hybrid
3AT: 3-Amino-1,2,4-triazole
