# Supplementary material for: RcMYB8 enhances salt and drought tolerance in rose (Rosa chinensis) by modulating RcPR5/1 and RcP5CS1
Source: Mol Hortic. 2024 Jan 29;4:3. doi: 10.1186/s43897-024-00080-9 (PMC10823735; doi:10.1186/s43897-024-00080-9)
Supplement: Supplementary file 1 — Additional file 1: Supplementary Figure S1. Domain structure and sequence alignment of RcMYB8 and R2R3 MYB proteins from other species. Supplementary Figure S2. Silencing of RcMYB8 decreased salt tolerance in rose leaves. Supplementary Figure S3. Relative expression of RcMYB8 in VC and pSuper: RcMYB8. Supplementary Figure S4. Silencing of RcMYB8 decreased drought tolerance in rose leaves. Supplementary Figure S5. Expression patterns of genes related to ROS scavenging and proline synthesis (A), pathogenesis-related protein 5 (B), and Na+, K+, Ca2+ transporter (C) under drought and salt stress treatments. Supplementary Figure S6. Sequence information of the RcPR5/1 promoter. Supplementary Figure S7. Self-activation detection of pAbAi-RcPR5/1 in yeast. Supplementary Figure S8. Schematic of the effector and reporter vector of RcMYB8 and RcPR5/1. Supplementary Figure S9. Domain structure and sequence alignment of RcP5CS1 with other plant P5CS proteins. Supplementary Figure S10. Expression profiles of RcP5CS1 under drought conditions at indicated time points. Supplementary Figure S11. Sequence information of the RcPR5/1 promoter. Supplementary Figure S12. Self-activation detection of pHis-RcP5CS1 in yeast. Supplementary Fig. S13. Schematic of the effector and reporter vector of RcMYB8 and RcP5CS1. [file 43897_2024_80_MOESM1_ESM.doc]

# Supplementary figures


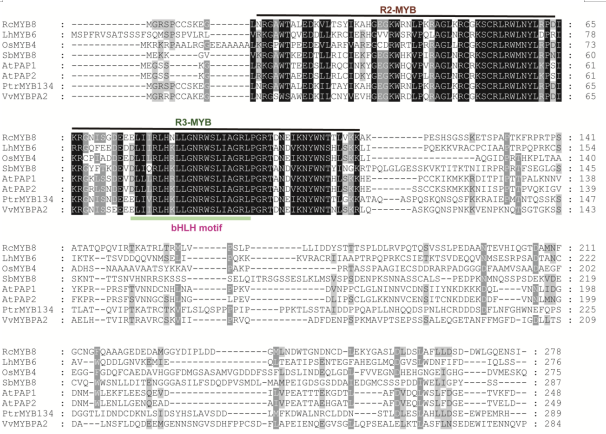


**Supplementary Figure S1** Domain structure and sequence alignment of RcMYB8 and R2R3 MYB proteins from other species.

**
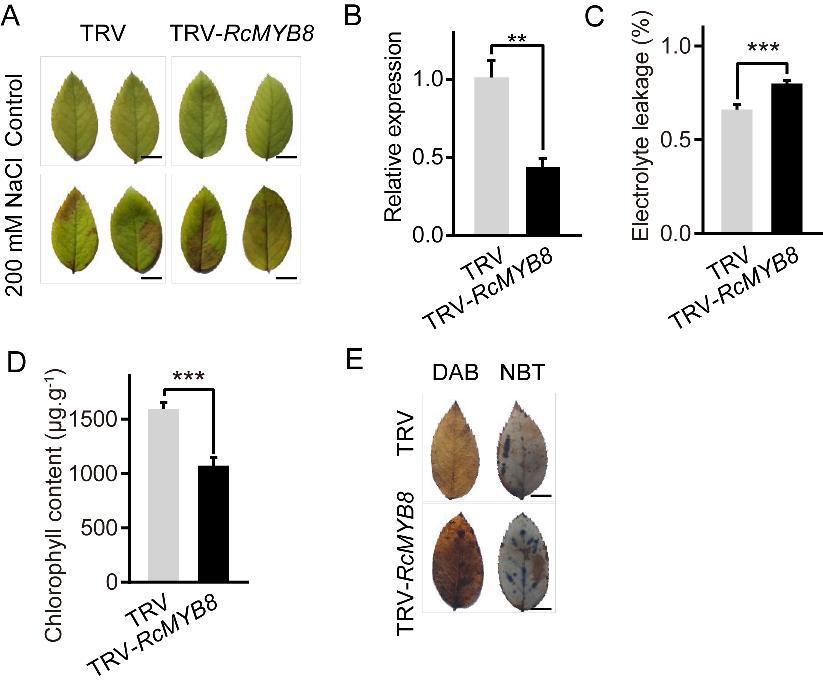
**

**Supplementary Figure S2 Silencing of *RcMYB8* decreased salt tolerance in rose leaves.**

(A) Phenotype of *RcMYB8*-silenced leaves under salinity stress. Scale bar: 1 cm.

(B) Relative expression of *RcMYB8* in TRV and TRV-*RcMYB8*. *RcUBI2* was used as an internal control of three biological replicates. Data represent the mean ± SD (n=3). **P*<0.05, *t*-test; ***P*<0.01, *t*-test.

(C and D) Electrolyte leakage and chlorophyll content of leaves in TRV and TRV-*RcMYB8* under salinity stress. Data represent the mean ± SD (n=3). **P*<0.05, *t*-test; ***P*<0.01, *t*-test.

(E) DAB and NBT staining of TRV and TRV-*RcMYB8* under salinity stress. Scale bar: 1 cm.


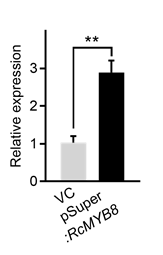


**Supplementary Figure S3** Relative expression of *RcMYB8* in VC and pSuper:*RcMYB8*. *RcUBI2* was used as an internal control of three biological replicates. Data represent the mean ± SD (n=3). **P*<0.05, *t*-test; ***P*<0.01, *t*-test.


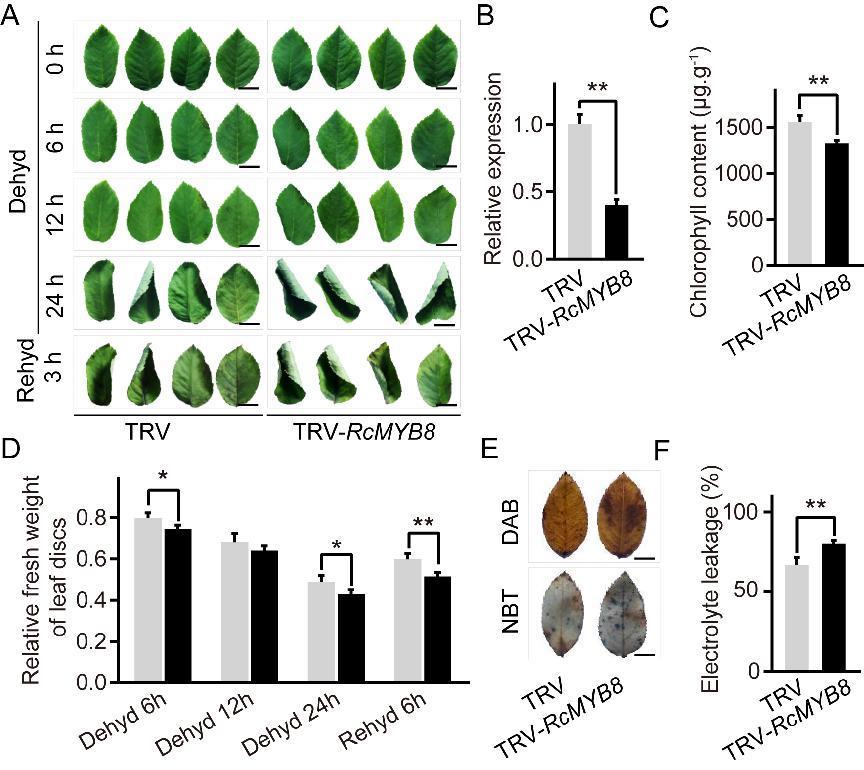


**Supplementary Figure S4** Silencing of *RcMYB8* decreased drought tolerance in rose leaves.

1. Phenotype of TRV and TRV-*RcMYB8* leaves in response to drought stress. Scale bar: 1 cm.
2. Relative expression of *RcMYB8* in TRV and TRV-*RcMYB8*. *RcUBI2* was used as an internal control of three biological replicates. Data represent the mean ± SD (n=3). **P*<0.05, *t*-test; ***P*<0.01, *t*-test.
3. Chlorophyll content differences between TRV and TRV-*RcMYB8* leaves.
4. Relative fresh weight of leaf discs between TRV and TRV-*RcMYB8* leaves.
5. Diaminobenzidine and nitroblue tetrazolium staining of TRV and TRV-*RcMYB8* leaves under dehydration 12 h. Scale bar: 1 cm.
6. Electrolyte leakage rate between TRV and TRV-*RcMYB8*. Data are mean with SD (n=3) of three independent experiments. Statistically significant differences (**P* ≤ 0.05; ***P* ≤ 0.01) as determined by *t*-test.


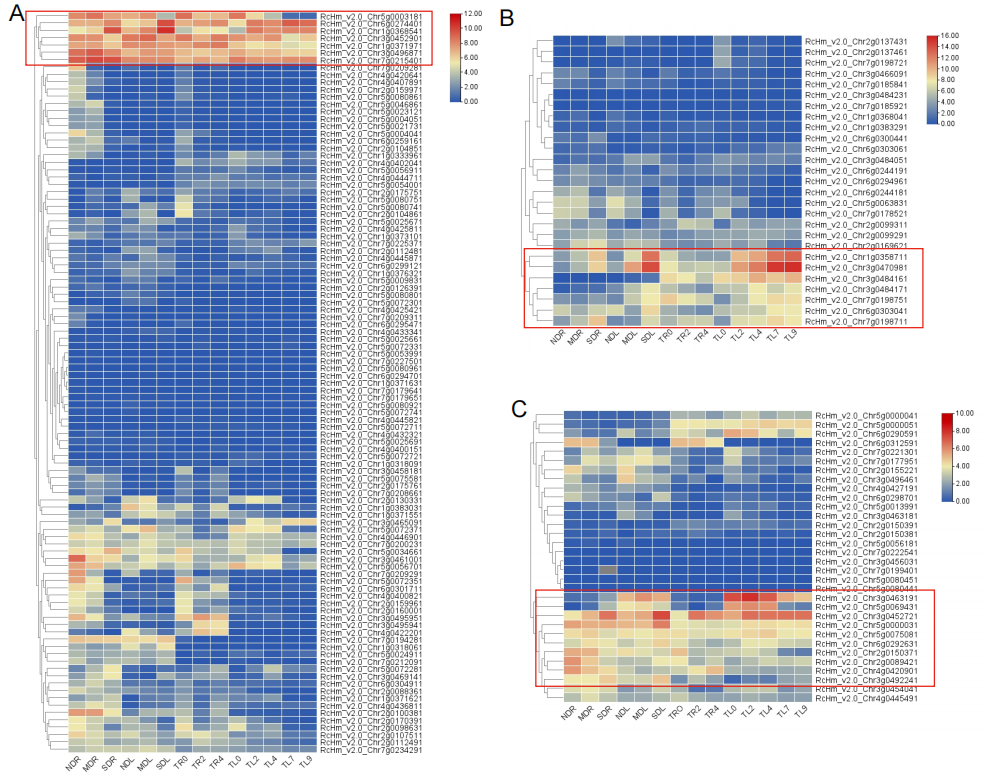


**Supplementary Figure S5** Expression patterns of genes related to ROS scavenging and proline synthesis(A), pathogenesis-related protein 5(B), and Na^+^, K^+^, Ca^2+^ transporter(C) under drought and salt stress treatments. The red box indicates genes that have the same expression pattern as *RcMYB8* under drought and salt stress. ND, MD, and SD. Represent controlled, moderate, and severe drought stress, respectively; T0, T2, T4, T7, and T9 represent 200 mM NaCl treatment for 0, 2, 4, 7, and 9 days, respectively; L, and R represent leaves and roots, respectively. The drought and salt stress treatments selected for this analysis were defined as described in (Li et al. 2021) and (Tian et al. 2018), respectively. The color code on the right represents the expression value; Blue indicates low transcript abundance, and red indicates high transcript abundance.

-1785 gtcgccctttggctgctaagcctgtgacttggtagataatggatcatattattttacttgatcgagattggtatttgataagttgagaca

-1695 ttttaaaaggggaaaaaatcttcttcagattaagaaatgtcgagtagttgttttctggtgtaaccatatttccaggaacgagttctactt

-1605 gtttgaatattcttaagcagttggataaataatcatatgtgggttttcaggcacagtcggggaagttgaatctaaccttaactatatttt

**P4**

-1515 cttgctgttctacttaccttgtttattatagtaactggtcaactaagcagaagaattttggtggtattgttgaccttgctatgaattaac

-1425 cagtagcaagcaaaatgtacaagaaacgtgtgctaattgccatagaaaacgcaaaagcccaaaaagactgctgaggctgctagccgcaat

-1335 tgtcatacttcaattccggcagccgtactattcacataagagaagcctagtgactacaatctgaaaaatgtgtttcaacatgaaattaaa

-1245 aaagagacacgtattccgggcgaatagcgtcatgctattttgctgataaatgtgatgtggttcaagaaatgagaatggtgagtagagttt

-1155 ctaactactgaccatgtactttcattaccctttgtatatgctttctgttgaattgcaatttcggggttttgattcgccttgaattagaag

-1065 tgtcgttcaggactagtttacacttgacagcttcatatttgtgtaattgatcaaagtcccttccaagcgaaaaagccagtcgggagtcgc

**P3**

-975 aagtttcataacttgtttgtgttggatttttcatggtcatgtcgtacacgatcaatattgtgttcttgtctagatatcatcagatatttt

-885 taggctatgttaacaatgactcgtatttgaggtttagaattgcttccttcctacatatgatgccataaccatattaactgatgagtggac

**P2**

-795 cacataaaataaatgctgcattagtcggttggaacctgcaaacctcctgtgcaaacccctttttatttcggataaaaatgttgatcctca

-705 ctatttttgtgaattctgaagtgttgtaaatatgatttaaaaatggaaaggaagaccttacgcctcttgtcctatcggagtgcctaatgg

-615 ttttacacatttacttgcttgtgaagttgggctatttagaattggaggctatattcctggggccttcgctgaaaatagttgagatgtgta

-525 agccctgttttttcaaataatggaagaacaaaactacaaaagtgtcaatgttgatgagaaaaagaactaaaaagaaattctgttttcttt

**P1**

-435 tcaatatgaatcgagatattttctcattttgtacatcttaatctgcatatcaaacgaggctctaatctaacatagcgactaggcaaaaca

-345 tataacttttctgtgctgctaggcgcgaatcggtgaaagaaaccatcttggactctgcctgctgctataactgttctgaccaaaataacc

-255 ttgcagctagggacttgattaattatggtgaaaagattttaacaaagggccctcaccaaaatcagagactcttccgttataaatacacaa

-165 tcctccagaccttttaagaacctctctcacctcccctctgccctctcctcctccgcacaatattgctgcaactgaaggacaccacccact

-75 tgacatttcctcctccattctcgatcaccagctagctagctagctaccagcagagctcaaaagggcgacacgcgaATG

**Supplementary Figure S6** Sequence information of the *RcPR5/1* promoter. The orange underline represents the sequence of P1. The yellow underline represents the sequence of P2. The green underline represents the sequence of P3.The blue underline represents the sequence of P4.Two types of MYB binding site are highlighted in yellow (TAACCA), green (MBSI sequence(C(A/C/G/T)GTT(A/G)).


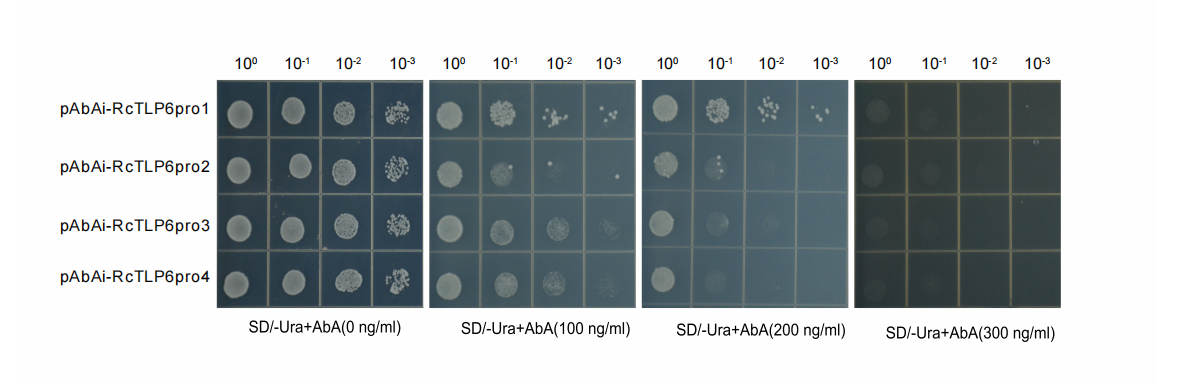


**Supplementary Figure S7** Self-activation detection of pAbAi-*RcPR5/1* in yeast. Yeast cells were diluted with distilled water (10^−1^ to 10^−3^) and grown on SD/−Ura medium containing 0, 100, 200, 300 ng/mL Aureobasidin A (AbA).


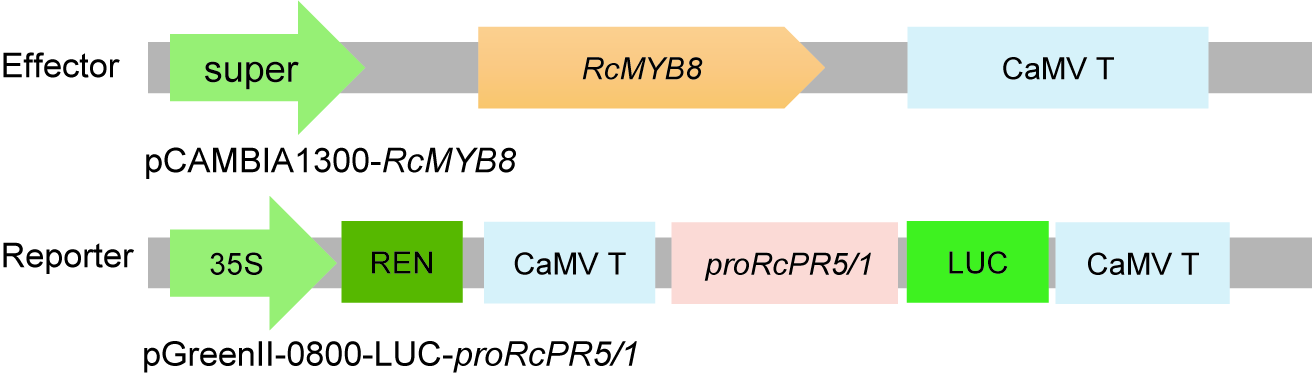


**Supplementary Figure S8** Schematic of the effector and reporter vector of *RcMYB8* and *RcPR5/1*. The effector is generated by recombining the *RcMYB8* gene into pCAMBIA 1300 vector. The promoter fragment of *RcPR5/1* (*RcPR5/1*-P2 and *RcPR5/1*-P4) was cloned into pGreenII 0800-LUC vector as a report construct.

**Supplementary**
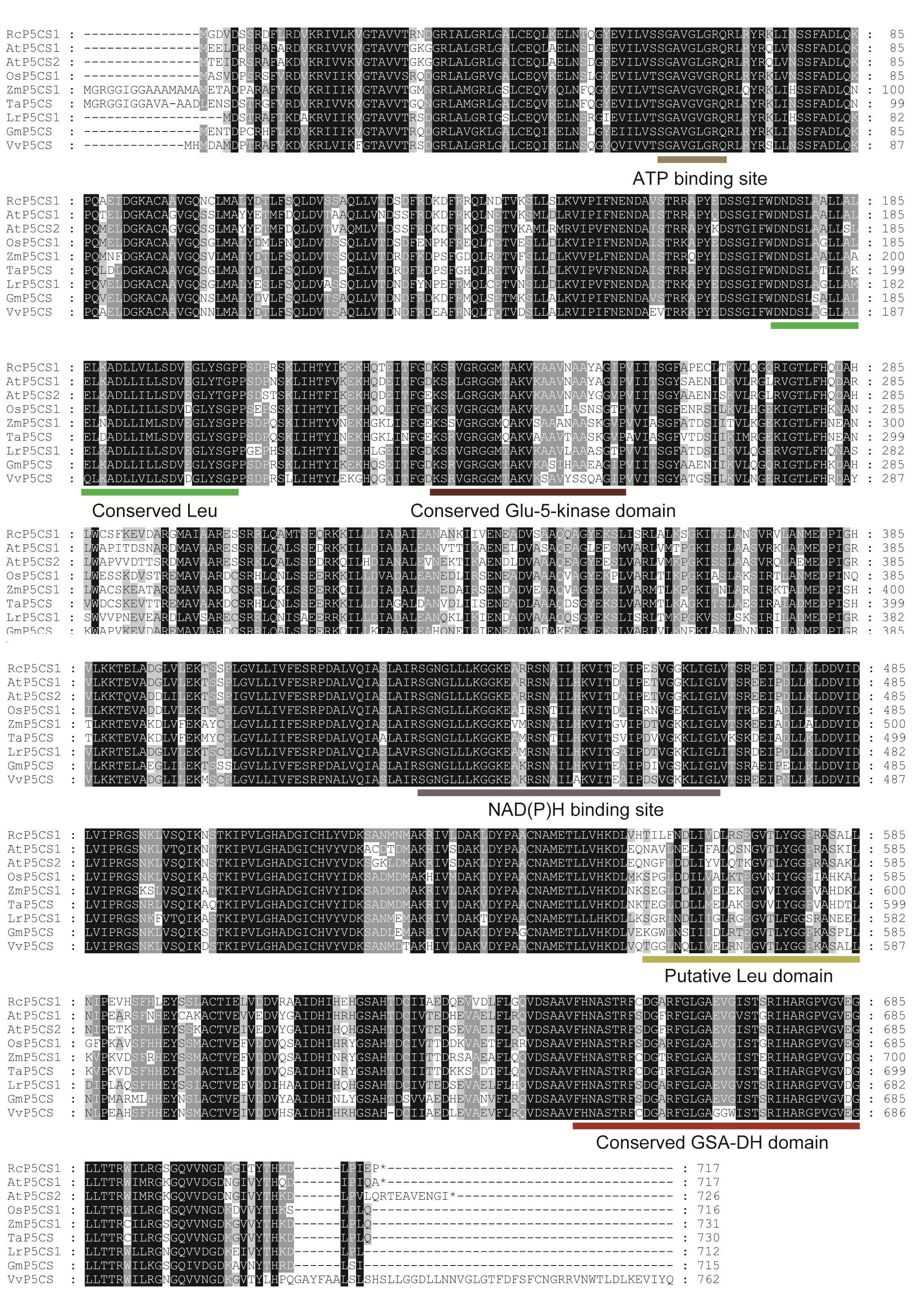
**Figure S9** Domain structure and sequence alignment of *RcP5CS1* with other plant P5CS proteins. Different color lines indicate ATP binding site, conserved Leu domain, conserved Glu-5-kinase domain, NAD(P)H binding site, putative Leu domain, and conserved GSA-DH domain, respectively.


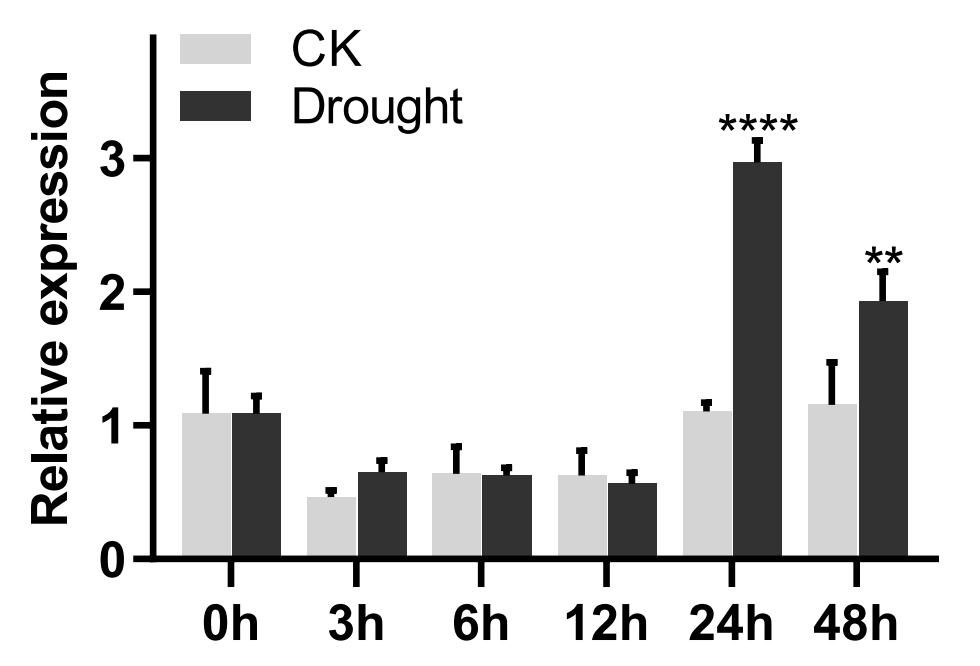


**Supplementary Figure S10** Expression profiles of *RcP5CS1* under drought conditions at indicated time points. The data originated from the three biological replicates of each treatment. Data are mean with SD (n=3) of at least three independent experiments, each with more than three plants. Statistically significant differences (*, *P* ≤ 0.05; **, *P* ≤ 0.01, ***, *P* ≤ 0.001, ****, *P* ≤ 0.0001) as determined by *t*-test.

-2000 tctagagattaacacgatcatagttgggagatagtttaggtcaagacaagaaataatcatggtccaaaatgacacgtagtgttacccttt

-1910 agacctaacgacaataatcggattagataattgtggtgttctgaaaacaatctgctttcgtaatcaaattattgatgcagcagcgtcggt

-1820 ttgcttcaattttgacacctatttcaaaaggtgacattgacatctcctgaacagttgaatataattattcgagttcaatcagaacagtat

-1730 ttatatcgacacgtagaggaaattgtgtctggtgatttcagtttaatacttttaaatcgtgtactttcgtttcttattggtggtcgttag

-1640 tgaaatgctttctttcttcatttttaattagttacaaaattgaaacaatgtgaatgtgattatgtagagatgagcatgggagatgccgtt

-1550 tagattgacaattctatgtttaaagtcatgatgtaattataaagtgtccaactttcacgagagtgaaaggatgactcctctaaaacaaga

-1460 aacttttacactaaagattataacttgacaccattattgactgatagcttgttgtggtatgatcactttggtatggatgcttgatttttg

-1370 ttttcttgtgtgattaatttagtttccttccggttgatttgagttgtgttgttagtaattgatctcttccttgccatcataggcgtttat

**P6**

-1280 tttgacacttgtaccttgtatactttgtattcaccttaacactgaatccaaagaagacaaagactcgccccacaacatctagtatcagta

-1190 agggtaaagtcttggtaggttcttactaaagtctccattggtacaagggaaaggtagaagaactagtgaactacgcttgaagtttagctc

-1100 tctctaagctagtgaaggatcagttgaagagaagtcaaggaatggatcaacatcataaacccaccaccactagtgctaaaacgcgcataa

-1010 tcacaagtagtgagacgattcgcactgtagcttcttcacaactaaagagagtagttgtcttctctgggtttttccccactactcagctat

-920 catcaacaaaatttatctccattccattgctaattaacaataataaaaagtagtattttgatacatataatatgctcttttgaaacataa

-830 aacatacacacacaaaacatgtataacacatatatgtttattattgatttttatttattcaaaacaattgtctggttgcggatcgtgaca

-740 cagttaatgagagaatacgaatcttttgggtaaaaaatgtattatcttttctcctaattatagaacaaatctgattagcctataaattat

-650 atcactcttaatttttcaaaattcttttaaaactcaagatttcgattcttcgtttctttgaatgtgtgatgctttacctagtaatgaata

-560 gataaagaagttttcctttataaaaaagagcatattcacgaataatactcatgatttatcactaaccaagaacttgtctcgtacttaatt

-470 agcaaatcttattagtctctacatgatttagggggtttggctgtctgacaatctgatcaaaagccacatgacacggatctttagcatctt

**P5**

-380 taactctcgagtaacttcgtcacttcctcttgtcttgctgtccacttaaccaggaagtcgccccgccgcctatttaactattctttacac

-290 tcacatccatgccactagtttaatccctcacctctccacccaataaccacccaccacctcatctctccctctccgatttcccataatgcc

-200 ctccactccggcgtcccattggtctgtgaccgcagcagcctgactatttgtaccctccagggccgcagttttagcgcaaacaacggccgc

-110 attccgcttccttacgaaaaaccctatcaaagaaagaaagaaaagtagcagacgcaccgccgagtcgactcgttcatttcgggaccgagt

-20 tttgactcgccaacgcagctATG

**Supplementary Figure S11** Sequence information of the *RcP5CS1* promoter. The blue underline represents the sequence of P5.The red underline represents the sequence of P6. MYB binding site are highlighted in yellow (TAACCA)


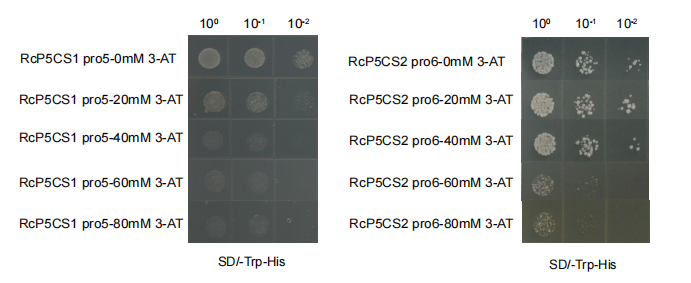


### Supplementary Figure S12 Self-activation detection of pHis-*RcP5CS1* in yeast. Yeast cells were diluted with distilled water (10^−1^ to 10^−2^) and grown on SD/−Trp-His medium containing 0, 20, 40, 60, 80 mM [**3-Amino-1,2,4-triazole**](https://www.so.com/link?m=bS0MAC7rR6OtE2/SneLd4nD1Y4u3U1uE29RbGrR4rEDClO8JFqc3M3AkyCi3hLYvDXW6M0zrHG9CLTgJfcm1qBnR9ZEafoGB6/RJYeaRXTdy/BQS9icVNb+81ZJNIaNxBVuUyb+B2QmM4Vdkkg8ocuQs9glKeC7NzZ0S9TrVq2WK8HLVbRWlJkugzagDsBfGvAmgJzKYB+R/euY121+SyW+0vtJEusKaZ4/fDXIRGPXnx3uuDZeiSpA2AgJxteJNAP2SBqwfT4mA=) (3-AT).

**
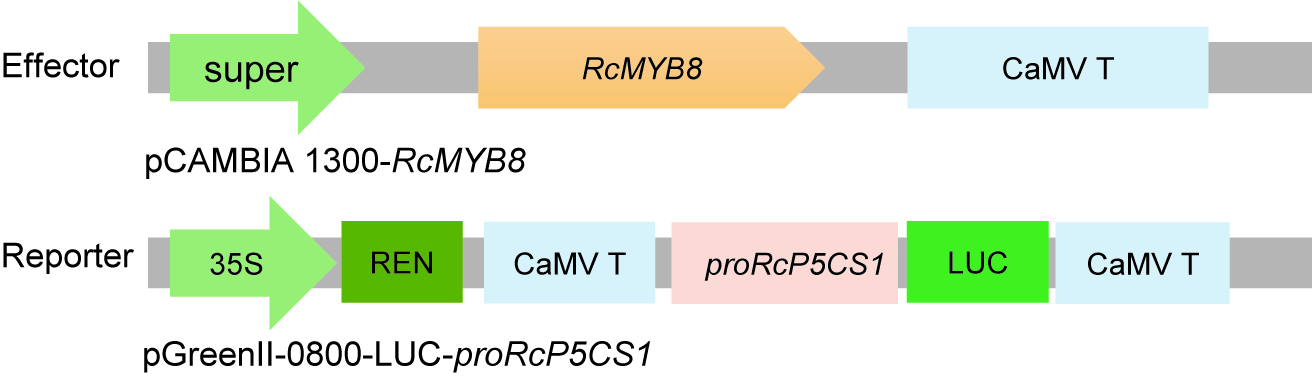
**

**Supplementary Figure S13** Schematic of the effector and reporter vector of *RcMYB8* and *RcP5CS1*. The effector is generated by recombining the *RcMYB8* gene into pCAMBIA 1300 vector. The promoter fragment of *RcP5CS1* was cloned into pGreenII 0800-LUC vector as a report construct.

# References

Li W, Fu L, Geng Z, Zhao X, Liu Q, Jiang X. Physiological characteristic changes and full-Length transcriptome of rose (*Rosa chinensis*) roots and leaves in response to drought stress. Plant Cell Physiol. 2021;61:2153-2166.

Tian X, Wang Z, Zhang Q, Ci H, Wang P, Yu L, et al. Genome-wide transcriptome analysis of the salt stress tolerance mechanism in *Rosa chinensis*. PLoS One. 2018;13:e0200938.
