# Supplementary material for: RcMYB8 enhances salt and drought tolerance in rose (Rosa chinensis) by modulating RcPR5/1 and RcP5CS1
Source: Mol Hortic. 2024 Jan 29;4:3. doi: 10.1186/s43897-024-00080-9 (PMC10823735; doi:10.1186/s43897-024-00080-9)
Supplement: Supplementary file 2 — Additional file 2: Supplementary Table S1. The primers sequences used in this study. Supplementary Table S2. List of genes used in phylogenic analysis of RcMYB8. Supplementary Table S3. List of accession numbers in this study. [file 43897_2024_80_MOESM2_ESM.doc]

# Supplementary tables

**Table S1** The primers sequences used in this study

| **Name** | **Forward** | **Reverse** |
| --- | --- | --- |
| For gene cloning in rose | | |
| *RcMYB8* | ATGGGGAGGAGTCCATGCT | TTAGATACTATTTTCTTGGCCCAG |
| *RcP5CS1* | ATGGGTGACGTGGATAGTTCTCG | TTAGGGCTCAATTGGGAGGTCC |
| For construction of silencing expression vector | | |
| TRV-*RcMYB8* | gtgagtaaggttaccgaattcCCCATCACTCCCACTTCTAATCG | gagacgcgtgagctcggtaccAGGAATCTAAATCCAATGAAGCACC |
| TRV-*RcP5CS1* | cgggatccgtccCATTGGTCCGTGA | cggaattcacagCTGCACATGCCTTC |
| For construction of transactivation activity vector | | |
| BD-RcMYB8 | aggacctgcatatggccATGGATGGGGAGGAGTCCATGCTG | atgcggccgctgcaggtcgacTTAGATACTATTTTCTTGGCCCAGC |
| For construction of overexpression vector | | |
| pSuper:RcMYB8 cgactctagtctagaaagcttATGGGGAGGAGTCCATGCTG *cctcgcccttgctcaccatgg*TTAGATACTATTTTCTTGGCCCAGC | | |
| For PCR detection of transgenic callus | | |
| pSuper:*RcMYB8* | tagtctagaaagcttctgcagATGGGGAGGAGTCCATGCTG | cactagtatttaaatgtcgacGATACTATTTTCTTGGCCCAGCC |
| For yeast vector construction in rose | | |
| AD-RcMYB8 | gtaccagattacgctcatatgATGGGGAGGAGTCCATGCTG | atgcccacccgggtggaattcTTAGATACTATTTTCTTGGCCCAGC |
| Pro*-*RcPR5/1 | TGGCTGCTAAGCCTGTGACTTG | TTGAGCTCTGCTGGTAGCTAGCTG |
| pAbAi -*RcPR5/1*-P1 | ccaaagcttgaattcgagctcATTGACTAATAATTCTAACACCAACCTCC | agcacatgcctcgaggtcgacGCCCTTTTTCTGATAACTGATTAGTATAC |
| pAbAi -*RcPR5/1*-P2 | ccaaagcttgaattc*gagctc*ATCATCAGATATTTTTAGGCTATGTTAACA | agcacatgcctcgag*gtcgac*AATAGTGAGGATCAACATTTTTATCCG |
| pAbAi -*RcPR5/1*-P3 | ccaaagcttgaattc*gagctc*TGCTTTCTGTTGAATTGCAATTTC | agcacatgcctcgag*gtcgac*TGGAAGGGACTTTGATCAATTACA |
| pAbAi -*RcPR5/1*-P4 | ccaaagcttgaattc*gagctc*GTTGTTTTCTGGTGTAACCATATTTCC | agcacatgcctcgag*gtcgac*CTGTGCCTGAAAACCCACATATG |
| Pro-*RcP5CS1* | AACACGATCATAGTTGGGAG | AGCTGCGTTGGCGAGTC |
| pHIS-*RcP5CS1*-P5 | gactcactatagggcgaattcTTAGCCTATAAATTATATCACTCTTAATTTTTC | gattcgcgaacgcgtgagctcAGCTGCGTTGGCGAGTCA |
| pHIS-*RcP5CS1*-P6 | gactcactatagggcgaattcAACACGATCATAGTTGGGAGATAGTT | gattcgcgaacgcgtgagctcTCAGATTTGTTCTATAATTAGGAGAAAAGA |
| For dual luciferase vector construction in rose | | |
| *RcPR5/1*-P2:LUC | gtcgacggtatcgataagcttATCATCAGATATTTTTAGGCTATGTTAACA | agtggatcccccgggctgcagAATAGTGAGGATCAACATTTTTATCCG |
| *RcPR5/1*-P4:LUC | gtcgacggtatcgataagcttGTTGTTTTCTGGTGTAACCATATTTCC | agtggatcccccgggctgcagCTGTGCCTGAAAACCCACATATG |
| *RcP5CS1*-P5:LUC | gggccccccctcgaggtcgacTTAGCCTATAAATTATATCACTCTTAATTTTTC | caggaattcgatatcaagcttAGCTGCGTTGGCGAGTCA |
| For Electrophoretic Mobility Shift Assay | | |
| PGEX-RcMYB8 | ccgcgtggatccccggaattcATGGGGAGGAGTCCATGCTG | gatgcggccgctcgagtcgacTTAGATACTATTTTCTTGGCCCAGC |
| For RT-qPCR in rose | | |
| qRT-*RcMYB8* | ATGGGGAGGAGTCCATGCT | TTAGATACTATTTTCTTGGCCCAG |
| qRT-*RcP5CS1* | CTTTTGAGTGATGTTGAGGGC | CCCTCTTCCCACTCTAGATTTG |
| qRT-*RcPR5/1* | CACCATTACCTTCTGTCCCTC | TTGTACTGTTTATCTCCGGCG |
| qRT-*RcPR5/2* | GCTACGATCACCTTCACCAAC | TTTTGGCTCCCTGTGCTAG |
| qRT-*RcPR5/3* | AATGACACTCCCGAAACCTG | CCACCGAAGCATGTAAACAAG |
| qRT-*RcPR5/4* | CCCTGTACTGTGTTCAAGACTG | GGGTAACTGTAAGCATCCGG |
| qRT-*RcPR5/5* | CTCTACCTCACTTTCGCCTTC | CCCGTCAATGGTAACTGAGG |
| qRT-*RcPR5/6* | ACTCCACCTAATGAAAAGCCAG | GTAATAGCGTAGTTAGGTCCCC |
| qRT-*RcPOD1* | TTCCTGACCACAATGAGAGC | ACTTCTGGGTACAAACGGTG |
| qRT-*RcPOD2* | TTGTTCAGGGATGTGATGGG | AACTCAGGTTTGGAGGTGC |
| qRT-*RcSOD1* | ACCAGGCTTACATCACCAAC | GAAAATCGAGTGGTTGACATGAC |
| qRT-*RcSOD2* | CCGGACCACACTCTATCATTG | CTAAGCTCATGTCCACCCTTG |
| qRT-*RcSOD3* | CGACTGGCAGATTCCACTAAG | CCAATTATACCGCATCCAACTC |
| qRT-*RcCAT1* | TCAACTGTTATCCACGAGCG | CACCAAGTCAAAATTGCCCTC |
| qRT-*RcAKT1* | GCAGAATGAAGCACCAACAG | ACAGAGTACCCCAATTTCACC |
| qRT-*RcAKT2* | CCTCATTGCTCTCCGGTATTTG | CCCGACTCAACCTTATCCATAG |
| qRT-*RcCAX1* | CCATGTTTGTGATTCCCTTCTG | ACGGTGATAAAGAGTGTAGCAG |
| qRT-*RcCAX2* | GGCTTCAAACCAAGAACCATG | CATGGAATTTTCGAGATGAGCG |
| qRT-*RcCAX3* | AGAATAAGGAGCAACGGAGTG | AGAAACAAGAATCGGGTCGG |
| qRT-*RcCAX4* | GAGTGGCAATAGGGTCATCG | AGTGTGGCTGTCTCAAAGAG |
| qRT-*RcNHX1* | CCACCCTCTCCAAAATCAGTC | CCAGTAGCGATGTACAGTGTG |
| qRT-*RcNHX2* | GATCGAAACTATCTCACCCCG | GTAGAACTAGACACAGGCTCATC |
| qRT-*RcNHX3* | CCTCACCTACAGACTTCCATTTG | CCAAACACCAGAGCATACAAG |
| qRT-*RcNHX4* | CTCTCCCACATTACCTCAACC | TGCCCGCCAAGATACAAC |
| qRT-*RcUBI2* | CACAAGCACGCAAACCCTAT | GGAGCATGAGCCAAATGGAG |

**Table S2 List of genes used in phylogenic analysis of *RcMYB8*.**

| **Gene name** | **Gene ID** | **References** |
| --- | --- | --- |
| RcMYB8 | XP_024165025.1 |  |
| AtPAP1 | AAG42001 | Qiu et al.,2014 |
| AtPAP2 | AAG42002 | Li et al.,2018 |
| VvMYBA1 | BAD18977.1 | Xia et al.,2021 |
| SbMYB8 | KF008657.1 | Yuan et al.,2015 |
| LhMYB6 | BAJ05399 | Yamagishi et al.,2010 |
| OsMYB4 | D88620.1 | Pasquali et al.,2008 |
| IbMYB308 | CAA2964916.1 | Wang et al.,2022 |
| VvMYBPA2 | ACK56131 | Terrier et al.2009 |
| VvMYBPA1 | CAJ90831.1 | Bogs et al.,2007 |
| PtrMYB134 | ACR83705.1 | Mellway et al.2009 |
| AtMYB96 | *AJ011669.1* | Seo et al.,2011 |
| GhMYB36 | ASH96785.1 | Liu et al.,2022 |
| AtMYB2 | AEC10812.1 | Abe et al.,1997 |
| ZmMYB48 | XM_008666380.2 | Wang et al.,2017 |
| AtMYB44 | Q9FDW1.1 | Jung et al.,2008 |
| OsMYB3R-2 | NP_001393248.1 | Ma et al.,2009 |
| TaMYB3R-1 | HQ236494.1 | Cai et al.,2011 |
| AtMYB3R-1 | AF151646 | Haga et al.,2007 |
| AtMYB3R-4 | Q94FL9.1 | Haga et al.,2007 |
| OsMYB3R-1 | AJ430051.1 |  |
| AtMYB4R | AY033827 |  |
| PbMYB4R | KX272615.1 |  |
| AtLHY | Q6R0H1.2 | Mizoguchi et al.,2002 |
| AtCCA1 | P92973.1 | Mizoguchi et al.,2002 |
| GmMYB118 | NP_001235909.2 | Du et al.,2018 |
| AtMYBD | AAP21221.1 | Nguyen et al.,2016 |
| GhMYB176 | NP_001236048.2 | Yi et al.,2010 |
| HvMCB1 | CAC24844.1 | Churin et al.,2003 |
| HvMCB2 | CAC24845.1 | Churin et al.,2003 |
| RcP5CS1 | PRQ59288.1 |  |
| RcP5CS2 | PRQ43126.1 |  |

| **Table S3** List of accession numbers in this study | |
| --- | --- |
| **Gene name** | **Gene ID** |
| *RcMYB8* | RcHm_v2.0_Chr6g0311681 |
| *RcPR5/1(RcTLP6)* | RcHm_v2.0_Chr6g0303041 |
| *RcP5CS1* | RcHm_v2.0_Chr1g0368541 |
| *RcP5CS2* | RcHm_v2.0_Chr3g0465091 |
| *RcPOD1* | RcHm_v2.0_Chr5g0003181 |
| *RcPOD2* | RcHm_v2.0_Chr6g0274401 |
| *RcSOD1* | RcHm_v2.0_Chr1g0371971 |
| *RcSOD2* | RcHm_v2.0_Chr3g0452901 |
| *RcSOD3* | RcHm_v2.0_Chr3g0496871 |
| *RcCAT1* | RcHm_v2.0_Chr7g0215401 |
| *RcPR5/2* | RcHm_v2.0_Chr3g0470981 |
| *RcPR5/3* | RcHm_v2.0_Chr1g0358711 |
| *RcPR5/4* | RcHm_v2.0_Chr7g0198751 |
| *RcPR5/5* | RcHm_v2.0_Chr3g0484171 |
| *RcPR5/6* | RcHm_v2.0_Chr3g0484161 |
| *RcAKT1* | RcHm_v2.0_Chr2g0089421 |
| *RcAKT2* | RcHm_v2.0_Chr3g0492241 |
| *RcCAX1* | RcHm_v2.0_Chr2g0150371 |
| *RcCAX2* | RcHm_v2.0_Chr3g0463191 |
| *RcCAX3* | RcHm_v2.0_Chr5g0069431 |
| *RcCAX4* | RcHm_v2.0_Chr5g0075081 |
| *RcNHX1* | RcHm_v2.0_Chr3g0452721 |
| *RcNHX2* | RcHm_v2.0_Chr5g0000031 |
| *RcNHX3* | RcHm_v2.0_Chr5g0000051 |
| *RcNHX4* | RcHm_v2.0_Chr6g0292631 |
| *RcUBI2* | RcHm_v2.0_Chr1g0359561 |

References

Abe H, Yamaguchi-Shinozaki K, Urao T, Iwasaki T, Hosokawa D, Shinozaki K. Role of *Arabidopsis MYC* and *MYB* homologs in drought- and abscisic acid-regulated gene expression. Plant Cell. 1997;9(10):1859-1868*.*

Bogs J, Jaffé FW, Takos AM, Walker AR, Robinson SP. The grapevine transcription factor *VvMYBPA1* regulates proanthocyanidin synthesis during fruit development. Plant Physiol. 2007;143(3):1347-61.

Cai H, Tian S, Liu C, Dong H. Identification of a *MYB3R* gene involved in drought, salt and cold stress in wheat (*Triticum aestivum* L.). Gene. 2011;485(2):146-152.

Churin Y, Adam E, Kozma-Bognar L, Nagy F, Börner T. Characterization of two Myb-like transcription factors binding to *CAB* promoters in wheat and barley. [Plant Mol Biol](https://www.so.com/link?m=ue4ZbDkLCwWgOo1yIk3vCC4TkmE9LGrld9ceIKIht06v93CkkW7KPYc79d69DqTW6a401xtrmuc9wwbvC/s6TihK+7AQLt2iojCx6ZQDE2nw4uAPf245IPJm+3R/GG+jDQLwVvDL0YeZ7FjElqsGABHyHM0lRF0fix4b9Cg==). 2003;52(2):447-62.

Du YT, Zhao MJ, Wang CT, Gao Y, Wang YX, Liu YW, et al. Identification and characterization of *GmMYB118* responses to drought and salt stress. BMC Plant Biol. 2018;18(1):320.

Haga N, Kato K, Murase M, Araki S, Kubo M, Demura T, Suzuki K, et al. R1R2R3-Myb proteins positively regulate cytokinesis through activation of KNOLLE transcription in *Arabidopsis thaliana*. Development. 2007;134(6):1101-1110.

Jung C, Seo JS, Han SW, Koo YJ, Kim CH, Song SI, et al. Overexpression of *AtMYB44* enhances stomatal closure to confer abiotic stress tolerance in transgenic Arabidopsis. Plant Physiol. 2008;146(2):623-35.

Li W, Fu L, Geng Z, Zhao X, Liu Q, Jiang X. Physiological characteristic changes and full-length transcriptome of rose (*Rosa chinensis*) roots and leaves in response to drought stress. Plant Cell Physiol. 2021;61(12):2153-2166.

Li N, Wu H, Ding Q, Li H, Li Z, Ding J, et al. The heterologous expression of *Arabidopsis PAP2* induces anthocyanin accumulation and inhibits plant growth in tomato. Funct Integr Genomics. 2018;18(3):341-353.

Liu T, Chen T, Kan J, Yao Y, Guo D, Yang Y, et al. The *GhMYB36* transcription factor confers resistance to biotic and abiotic stress by enhancing *PR1* gene expression in plants. Plant Biotechnol J. 2022;20(4):722-735.

Ma Q, Dai X, Xu Y, Guo J, Liu Y, Chen N, et al. Enhanced tolerance to chilling stress in *OsMYB3R-2* transgenic rice is mediated by alteration in cell cycle and ectopic expression of stress genes. Plant Physiol. 2009;150(1):244-56.

Mellway RD, Tran LT, Prouse MB, Campbell MM, Constabel CP. The wound-, pathogen-, and ultraviolet B-responsive *MYB134* gene encodes an R2R3 MYB transcription factor that regulates proanthocyanidin synthesis in poplar. Plant Physiol*.* 2009;150(2):924-41.

Mizoguchi T, Wheatley K, Hanzawa Y, Wright L, Mizoguchi M, Song HR, et al. *LHY* and *CCA1* are partially redundant genes required to maintain circadian rhythms in Arabidopsis. Dev Cell*.* 2002;2(5):629-41.

Nguyen NH, Lee H. MYB-related transcription factors function as regulators of the circadian clock and anthocyanin biosynthesis in Arabidopsis. [Plant Signal Behav](https://www.so.com/link?m=wgMKQa/fs9mjO36MLbqzjHL4SUSKvX2xqJuFkllW27ryuOr4wvOMCeK/faaE9LaUWnJdyjgpWO7yD8gjuqozzNIir+CpvMpLFr03ZuL8ilI1/UERw8C9DCdbfqHbtgMVyeBw7rQ+2cVmemR3MYSmyyXq1ixA=). 2016;11(3):e1139278.

Pasquali G, Biricolti S, Locatelli F, Baldoni E, Mattana M. *Osmyb4* expression improves adaptive responses to drought and cold stress in transgenic apples. Plant Cell Rep. 2008;27(10):1677-86.

Qiu J, Sun S, Luo S, Zhang J, Xiao X, Zhang L, et al. *Arabidopsis* *AtPAP1* transcription factor induces anthocyanin production in transgenic *Taraxacum brevicorniculatum*. Plant Cell Rep. 2014;33(4):669-80.

Seo PJ, Lee SB, Suh MC, Park MJ, Go YS, Park CM. The *MYB96* transcription factor regulates cuticular wax biosynthesis under drought conditions in *Arabidopsis*. Plant Cell. 2011;23(3):1138-52.

Terrier N, Torregrosa L, Ageorges A, Vialet S, Verriès C, Cheynier V, et al. Ectopic expression of *VvMybPA2* promotes proanthocyanidin biosynthesis in grapevine and suggests additional targets in the pathway. Plant Physiol. 2009;149(2):1028-41.

Tian X, Wang Z, Zhang Q, Ci H, Wang P, Yu L, et al. Genome-wide transcriptome analysis of the salt stress tolerance mechanism in *Rosa chinensis*. PLoS One. 2018;13(7):e0200938.

Wang C, Wang L, Lei J, Chai S, Jin X, Zou Y, et al. *IbMYB308*, a sweet potato R2R3-MYB gene, improves salt stress tolerance in transgenic tobacco. Genes (Basel). 2022;13(8):1476.

Wang Y, Wang Q, Liu M, Bo C, Wang X, Ma Q, et al. Overexpression of a maize *MYB48* gene confers drought tolerance in transgenic *Arabidopsis* plants. [J Plant Biol](https://www.researchgate.net/journal/Journal-of-Plant-Biology-1867-0725). 2017;60:612–621.

Xia H, Shen Y, Hu R, Wang J, Deng H, Lin L, et al. Methylation of *MYBA1* is associated with the coloration in "*Manicure Finger*" grape Skin. J Agric Food Chem. 2021;69(51):15649-15659.

Yamagishi M, Shimoyamada Y, Nakatsuka T, Masuda K. Two R2R3-MYB genes, homologs of Petunia *AN2*, regulate anthocyanin biosyntheses in flower tepals, tepal spots and leaves of asiatic hybrid lily. Plant Cell Physiol. 2010;51(3):463-474.

Yi J, Derynck MR, Li X, Telmer P, Marsolais F, Dhaubhadel S. A single-repeat MYB transcription factor, *GmMYB176*, regulates *CHS8* gene expression and affects isoflavonoid biosynthesis in soybean. Plant J. 2010;62(6):1019-34.

Yuan Y, Qi L, Yang J, Wu C, Liu Y, Huang L. Erratum to: A *Scutellaria baicalensis* R2R3-MYB gene, *SbMYB8*, regulates flavonoid biosynthesis and improves drought stress tolerance in transgenic tobacco. [Plant Cell, Tissue Org](https://link.springer.com/journal/11240)an Cult. 2015;120:961–972.
